# Inter- and intra-specific pan-genomes of *Borrelia burgdorferi* sensu lato: genome stability and adaptive radiation

**DOI:** 10.1186/1471-2164-14-693

**Published:** 2013-10-10

**Authors:** Emmanuel F Mongodin, Sherwood R Casjens, John F Bruno, Yun Xu, Elliott Franco Drabek, David R Riley, Brandi L Cantarel, Pedro E Pagan, Yozen A Hernandez, Levy C Vargas, John J Dunn, Steven E Schutzer, Claire M Fraser, Wei-Gang Qiu, Benjamin J Luft

**Affiliations:** 1 Institute for Genome Sciences, University of Maryland School of Medicine, Baltimore, Maryland 21201, USA; 2 Department of Pathology, Division of Microbiology and Immunology, University of Utah Medical School, Salt Lake City, Utah 84112, USA; 3 Department of Medicine, Health Science Center, Stony Brook University, Stony Brook, New York 11794, USA; 4 Department of Biological Sciences, Hunter College of the City University of New York, New York, New York 10065, USA; 5 Biology Department, Brookhaven National Laboratory, Upton, New York 11793, USA; 6 Department of Medicine, University of Medicine and Dentistry of New Jersey, New Jersey Medical School, Newark, New Jersey 07103, USA

**Keywords:** *Borrelia burgdorferi*, Lyme borreliosis, Pan-genome, Single-nucleotide polymorphisms, Phylogenetic tree, Genome evolution simulation

## Abstract

**Background:**

Lyme disease is caused by spirochete bacteria from the *Borrelia burgdorferi* sensu lato (*B*. *burgdorferi* s.l.) species complex. To reconstruct the evolution of *B*. *burgdorferi* s.l. and identify the genomic basis of its human virulence, we compared the genomes of 23 *B*. *burgdorferi* s.l. isolates from Europe and the United States, including *B*. *burgdorferi* sensu stricto (*B*. *burgdorferi* s.s., 14 isolates), *B*. *afzelii* (2), *B*. *garinii* (2), *B*. “*bavariensis*” (1), *B*. *spielmanii* (1), *B*. *valaisiana* (1), *B*. *bissettii* (1), and *B*. “*finlandensis*” (1).

**Results:**

Robust *B*. *burgdorferi* s.s. and *B*. *burgdorferi* s.l. phylogenies were obtained using genome-wide single-nucleotide polymorphisms, despite recombination. Phylogeny-based pan-genome analysis showed that the rate of gene acquisition was higher between species than within species, suggesting adaptive speciation. Strong positive natural selection drives the sequence evolution of lipoproteins, including chromosomally-encoded genes *0102* and *0404*, cp26-encoded *ospC* and *b08*, and lp54-encoded *dbpA*, *a07*, *a22*, *a33*, *a53*, *a65*. Computer simulations predicted rapid adaptive radiation of genomic groups as population size increases.

**Conclusions:**

Intra- and inter-specific pan-genome sizes of *B*. *burgdorferi* s.l. expand linearly with phylogenetic diversity. Yet gene-acquisition rates in *B*. *burgdorferi* s.l. are among the lowest in bacterial pathogens, resulting in high genome stability and few lineage-specific genes. Genome adaptation of *B*. *burgdorferi* s.l. is driven predominantly by copy-number and sequence variations of lipoprotein genes. New genomic groups are likely to emerge if the current trend of *B*. *burgdorferi* s.l. population expansion continues.

## Background

Lyme disease, caused by the spirochete bacteria *Borrelia burgdorferi*, has become the most common vector-borne disease in the United States and Europe [1]. The genome organization of the bacterium and the spectrum of clinical manifestations associated with Lyme disease have presented a number of research challenges. Lyme disease is frequently a multisystem infection, commonly affecting the skin, joints, and central nervous system in humans [2,3], yet at other times the symptoms are restricted to the skin. Although much of the attention on *B*. *burgdorferi* involves the disease in humans, there is a complex relationship between the microbe in its vector, the *Ixodes* tick, the animals it can infect, and the environment. For example, the prevalence of *B*. *burgdorferi* is associated with the geographic range and abundance of its host species rather than its tick (*Ixodes scapularis*) vector [4-6]. Although it has been found in many vertebrates in the United States, white-footed mice (*Peromyscus leucopus*) and eastern chipmunks (*Tamias striatus*) serve as major maintenance reservoirs of *B*. *burgdorferi*; however, there may be other preferred host species for different local strains [7,8]. In Europe, *B*. *burgdorferi* (as well as *B*. *garinii* and *B*. *afzelii* which also cause Lyme disease) is transmitted by *Ixodes ricinus* ticks [9] and is carried by a large variety of hosts including birds and small-to-medium sized mammals [10]. For many reasons Lyme disease remains a puzzling emerging disease [3,11].

Globally, the *B*. *burgdorferi* species complex [12], *B*. *burgdorferi* sensu lato (*B*. *burgdorferi* s.l.), is classified into different genomic groups or species (sometimes called “genospecies”) on the basis of their molecular phylogeny. These species differ by 1%-2% in the 16S ribosomal RNA sequences [13] and by about 9% in average genome sequences where the latter is known [14] (see below). The most common species in North America are *B*. *burgdorferi* sensu stricto (*B*. *burgdorferi* s.s.) in the northeast and north central parts of the United States and *B*. *bissettii* in California and the western United States [15-18]. The most common species in Europe are *B*. *garinii*[19], *B*. *afzelii*[20], *B*. *burgdorferi* s.s., *B*. *valaisiana* and *B spielmanii*[9,21]. *B*. *garinii*, *B*. *afzelii*, and *B*. *valaisiana* are also common in northern Asia, but *B*. *burgdorferi* s.s. is absent from eastern Asia [22]. The three species *B*. *burgdorferi* s.s., *B*. *garinii*, and *B*. *afzelii* are the most common pathogens of Lyme disease, and they are each associated with different clinical manifestations of chronic Lyme disease [3,23]. More recently *B*. *bissettii*, *B*. *lusitaniae*, *B*. *spielmanii* and *B*. *valaisiana* have been isolated from human patients [24-29]. A series of molecular genotyping assays using genome-wide sequence signatures and individual loci have found genetic differentiation between the two continental *B*. *burgdorferi* s.s. populations, a European origin of the North American populations, and a few shared genotypes between the European and North American populations perhaps caused by contemporary migrations [30-33]. Intriguingly, we find that genotypes characterized as highly pathogenic in humans are also the ones that have a broad host-species range, able to colonize both continents [34].

Intra-specific lineages of *B*. *burgdorferi* s.s. can be differentiated by 16S-23S ribosomal RNA spacer (IGS) and outer surface protein C gene (*ospC*) sequences [35-38]. It has been found that these different intra-specific lineages may be related to different levels of pathogenicity. For example, a particular restriction fragment length polymorphism (RFLP) in the *B*. *burgdorferi* IGS sequence and *ospC* type are associated with hematogenous dissemination in patients with early stage Lyme disease [39-41]. A significant number of *ospC* clonal types associated with invasive disease in humans have also been found in *B*. *afzelii* and *B*. *garinii*[42]; however, the *ospC* clonal types isolated from patients with invasive disease are not limited to those types [43]. These subtypes have been further subdivided using a broad range primer assay coupled with mass spectrometry [44]. However, none of these studies examined entire genomes in its entirety, so conclusions remain limited.

The overall objective of the study reported here was to develop an informative genome-wide picture of *B*. *burgdorferi* diversity, with the ultimate aim of understanding how variations in genomic composition may lead to variations in pathogenicity. Although this study did not examine the molecular basis of Lyme disease *per se*, we believe it will greatly enhance such studies in many ways. For example, conserved genome features should in principle be those likely to be most essential in the *Borrelia* life cycle, while variable ones might be those that are more important immunologically and ecotypically. The present study was undertaken to help the scientific community generate hypotheses about what genes are related to human disease or of ecologic importance in the life cycle of this pathogen. Furthermore, such studies should provide some insight as to whether there is potential for the non-Lyme disease causing species to serve as a reservoir of genetic diversity for those that cause Lyme disease.

## Results and discussion

### Harvesting the genomic diversity of the Lyme agent through selection of *Borrelia* strains for whole-genome shotgun sequencing

Research efforts aimed at deciphering the mechanisms of *Borrelia* pathogenicity have resulted in significant progress over the past few years [45]. However, the costs and difficulties for propagation of the bacteria in culture, the prolonged doubling time of *Borrelia*, in addition to the limited tools that are available for genetic manipulation, have presented a number of significant challenges. Therefore, rigorous genetic studies and biochemical approaches that require even moderate amounts of biological materials are expensive, technically difficult and slow. Because of recent dramatic reductions in the DNA sequencing costs, comparative genomics studies of *Borrelia* species are now a cost effective way to provide a firm foundation for the generation of new, informed, and testable hypotheses which would be difficult or impossible to formulate by other means.

**Figure 1:**
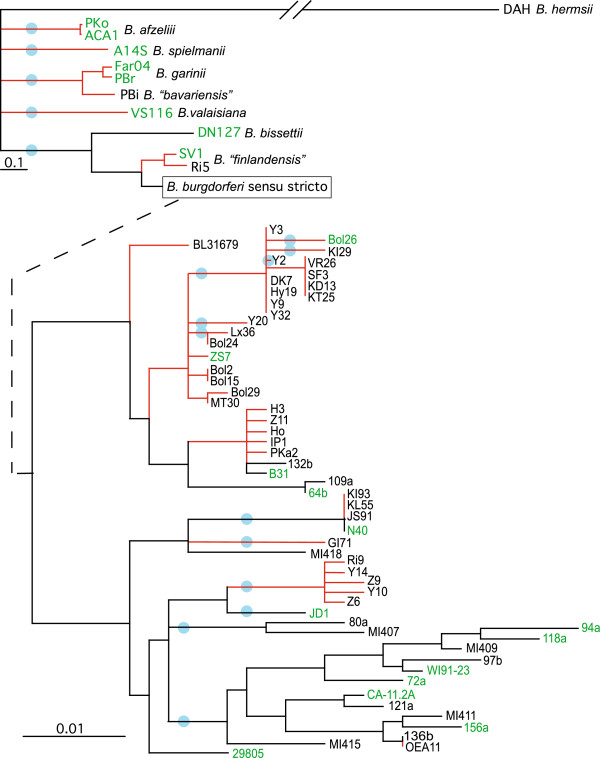
**MLST phylogenetic tree for 64 *Borrelia *isolates**, **representing the diversity of the Lyme agent and relapsing fever group.** A Bayesian tree of MLST data (constructed as described in Methods) is shown where the geographic provenance of isolates is represented by the color of their respective branches: black for North American isolates and red for European isolates. The dashed line indicates the attachment of the expanded *B*. *burgdorferi* s.s. tree below. We sequenced the genomes of those strains with green names. Scale bars for the top and bottom sections indicate distances in fractional change in nucleotide sequence. Blue circles mark branches which were merged since their bootstrap support is <90%. All other branches shown have bootstrap support ≥90% and the large majority have 100% support.

The *Borrelia* genus contains two major clades, one that includes the Lyme disease agents, and another that includes the relapsing fever agents, each of which contains numerous species [12,46,47]. Variation among *B*. *burgdorferi* isolates has most recently been analyzed informatively using MLST analyses [33,48,49]. In order to choose a panel of isolates for genome sequencing that maximized the represented *B*. *burgdorferi* s.s. genetic diversity, we MLST-typed 64 such isolates (Figure 1) that reside in different major groups based on rRNA IGS1 spacer sequence [35,38] and *ospC* sequence [35,36] while also attempting to include a variety of hosts and geographical regions. In addition, we included geographically diverse *B*. *burgdorferi* s.l. isolates, including several species that are not known to commonly cause Lyme disease. Twenty-two isolates were chosen for sequencing that included most of the major branches of this MLST tree (Table 1).

**Table 1:** ***Borrelia *** **isolates analyzed in this study**

**Species**	**Strain**	**rRNA IGS lineage**	***ospC *** **type**	**Genome status**	**No**. **of plasmids**	**GPID**^***a***^	**Isolation and sequence references**
*B*. *burgdorferi*	B31	1	A	Complete	21	3	[57,86,112]
	Bol26	3	S	Draft	13	19837	[34,50]
	ZS7	16	Bb	Draft	14	19839	[50,113]
	64b	3	Ba	Draft	18	28633	[50,114]
	297	2	K	Complete^b^	20	39123	[50,115]
	156a	12	Hb	Complete	20	19835	[50,114]
	WI-91-23	7	Ia	Draft	21	28627	[50,116]
	94a	8	U	Draft	14	20999	[50,114]
	JD1	24	C	Complete	20	39121	[50,117]
	CA-11.2A	19	Dd	Draft	12	28629	[50,118]
	118a	20	J	Draft	19	21001	[50,114]
	N40	9	E	Complete	17	39119	[50,119]
	72a	26	G	Draft	13	21003	[50,114]
	29805	6	M	Draft	15	28621	[50,120]
*B*. “*finlandensis*”	SV1	---	---	Draft	10	28631	[34,53]
*B*. *valaisiana*	VS116	---	---	Draft	11	19843	[52,121]
*B*. *bissettii*	DN127	---	---	Complete	16	29363	[52,122]
*B*. *afzelii*	PKo	---	---	Complete	17	68149, 17057	[51,54,123]
	ACA-1	---	---	Draft	14	19841	[51,124]
*B*. *garinii*	PBr	---	---	Draft	12	28625	[51,123]
	Far04	---	---	Draft	7	29573	[51,125]
*B*. “*bavariensis*”	PBi	---	---	Incomplete	(11)^c^	58125	[14,48,126]
*B*. *spielmanii*	A14S	---	---	Draft	13	28635	[52,127]

^a^Genome Project ID. Data from each genome can be retrieved directly from the NCBI Entrez Genome Project Database (http://www.ncbi.nlm.nih.gov/bioproject) using the Genbank Genome Project ID.

^b^For *B*. *burgdorferi* strain 297, all the plasmids have been identified and sequenced to closure. However, the chromosome of 297 was not sequenced.

^c^The complete sequences for all strain PBi plasmids have not yet been reported.

These isolates include 14 *B*. *burgdorferi* s.s. strains (in one of these, strain 297, the chromosome was not sequenced) which include six isolates from ticks (including ones from *Ixodes scapularis*, *I*. *pacificus* and *I*. *ricinus*), a wild bird isolate (from a Song Sparrow, *Melospiza melodia*), and seven isolates from human Lyme disease patients [50]. Within these s.s. isolates, considerable geographic distribution is also present, and it includes isolates from California, Wisconsin, New York, Massachusetts and Connecticut as well as Germany and Italy. In addition, eight other *B*. *burgdorferi* s.l. strains were chosen for sequencing, including two *B*. *afzelii* (Sweden and Germany), two *B*. *garinii* (Denmark and Germany) [51], single *B*. *bissettii* (California), *B*. *spielmanii* (The Netherlands) and *B*. *valaisiana* (Switzerland) isolates [52], and one isolate that may be an uncharacterized species (tick isolate SV1 from Finland) [53]. The latter seven isolates include human, bird (Atlantic Puffin, *Fratercula arctica*) and tick (*I*. *ricinus*) isolates. Table 1 lists the isolates whose genomes we analyze in this report along with their rRNA IGS and *ospC* types. In addition, the chromosome and some plasmid sequences have been reported for *B*. “*bavariensis*” strain PBi [14,54], so altogether the sequences of 22 chromosomes and 345 plasmid sequences have been reported from 23 *B*. *burgdorferi* s.l. genomes.

### *Borrelia* chromosomal diversity

Chromosomal sequences from the above thirteen strains of *B*. *burgdorferi* s.s. and nine other *Borrelia* s.l. isolates were aligned using the Mugsy genome aligner to compute a conserved chromosomal core sequence alignment which consisted of 843,710 bp of nucleotide sequence present in all chromosome sequences (see Methods for details). The difference between this value and the ~903,000 bp of the chromosomal constant regions (see below) is nearly all due to gaps between contigs in the draft chromosome sequences. In agreement with various less comprehensive methods [16,55], we find that the chromosomes of different isolates and species of *B*. *burgdorferi* s.l. are syntenic and conserved across nearly the entire length of the chromosome.

Length differences of up to about 20 kbp among *B*. *burgdorferi* chromosomes are due to different lengths of plasmid-like DNA sequences attached to their right ends ([56-58] and our unpublished analysis). The constant chromosomal region spans approximately 903 kbp located at the left end of the chromosome, and is delimited by type-strain B31 genes *b31*_*0001* through *b31*_*0843* (for gene description and nomenclature in this report, we use the nomenclature schema recommended by Casjens *et al*. [59] in which the isolate name in lower case precedes the number part of the GenBank locus tag; this naming strategy, unlike previous ones, distinguishes orthologues from different isolates. We furthermore recommend not using the strain specific prefix, *e*.*g*., “*b31*_”, when referring to a generic set of orthologues). In order to have consistent annotations across all the chromosomes sequenced, the B31 genome annotation was refreshed during this study and now predicts 815 genes in this region, which occupy 93.5% of the chromosome constant region (Accession Nos. AE000783-794 and AE001575-1584). We examined three of the *B*. *burgdorferi* chromosomes for open reading frame differences and disruptions, and found 18, 2 and 6 disruptions in strains B31, N40 and JD1, respectively (Additional file 1: Table S1). Some of the apparent disruptions in B31 are likely due to sequencing errors, since base calling and genome assembly were less accurate in 1997 when strain B31 was originally sequenced, There are very few obviously disrupted genes in the constant regions of these chromosomes.

The constant regions of the *B*. *burgdorferi* s.s. linear chromosomes are remarkably similar. Nucleotide differences between the thirteen chromosomes range from only 0.084% (strain ZS7 compared to Bol26) to 0.625% (strain 94a compared to 29805) (Table 2). Furthermore, only four indels larger than 30 bp are present. A 157-bp deletion appears to inactivate chromosomal gene *0021*, which is predicted to encode the tRNA modification enzyme that synthesizes queuosine in a number of the *B*. *burgdorferi* sequences, and variable numbers of tandem repeat sequences are present in three genes *0210*, *0546* and *0801* (details in Additional file 2: Table S2) (no strain indicator is included here in the names of orthologous gene sets; see [59] for gene nomenclature). Gene *b31*_*0210* encodes the surface protein Lmp1 that may be required for host serum resistance [60,61], and *b31*_*0546* and *b31*_*0801* encode a protein of unknown function and translation initiation factor 2, respectively (no other repeat-containing chromosomal genes were found with TandemRepeatsFinder [62]). In the *B*. *burgdorferi* s.s. chromosomes, the numbers of these repeats vary from 5 to 8, 3 to 5 and 10 to 12, respectively, in these three genes (Additional file 2: Table S2), so these variable number tandem repeat (VNTR) loci should be useful for lineage or epidemic tracking in *Borrelia*.

**Table 2:** **Comparison of *B***. ***burgdorferi *** **sensu lato chromosomes**

	***B. burgdorferi***												***B. "finlandensis"***	***B. bissettii***	***B. afzelii***		***B. spielmanii***	***B. "bavariensis"***	***B. garinii***		***B. valaisiana***	
**Isolate**	**ZS7**	**Bol26**	**64b**	**JD1**	**156a**	**118a**	**72a**	**CA-11.2A**	**94a**	**WI91-23**	**N40**	**29805**	**SV1**	**DN127**	**ACA-1**	**Pko**	**A14S**	**PBi**	**Far04**	**PBr**	**VS116**	
**B31**	0.309	0.298	0.259	0.469	0.488	0.511	0.515	0.506	0.525	0.519	0.572	0.553	1.719	5.051	7.114	7.117	7.671	7.230	7.262	7.221	7.299	
**ZS7**		0.084	0.321	0.522	0.515	0.549	0.551	0.554	0.578	0.541	0.599	0.582	1.750	5.070	7.076	7.079	7.657	7.228	7.260	7.219	7.301	
**Bol26**			0.312	0.512	0.504	0.540	0.541	0.541	0.566	0.533	0.586	0.571	1.751	5.068	7.080	7.082	7.658	7.227	7.262	7.220	7.299	
**64b**				0.471	0.488	0.498	0.501	0.504	0.515	0.512	0.575	0.577	1.720	5.052	7.112	7.115	7.670	7.240	7.266	7.224	7.299	
**JD1**					0.447	0.467	0.465	0.477	0.471	0.522	0.608	0.602	1.729	5.053	7.121	7.122	7.677	7.237	7.269	7.222	7.311	
**156a**						0.428	0.433	0.489	0.490	0.498	0.563	0.560	1.700	5.040	7.108	7.110	7.668	7.222	7.254	7.209	7.290	
**118a**							0.140	0.273	0.427	0.482	0.587	0.588	1.725	5.051	7.110	7.113	7.666	7.224	7.258	7.216	7.303	
**72a**								0.262	0.417	0.473	0.591	0.595	1.732	5.051	7.113	7.115	7.670	7.229	7.262	7.218	7.301	
**CA-11.2A**									0.437	0.479	0.584	0.593	1.729	5.053	7.114	7.116	7.678	7.231	7.260	7.220	7.308	
**94a**										0.529	0.591	0.625	1.746	5.065	7.130	7.131	7.678	7.242	7.273	7.230	7.317	
**WI91-23**											0.526	0.544	1.740	5.056	7.123	7.126	7.666	7.233	7.260	7.217	7.302	
**N40**												0.480	1.773	5.075	7.127	7.130	7.677	7.231	7.268	7.223	7.312	
**29805**													1.777	5.082	7.132	7.135	7.687	7.238	7.268	7.224	7.310	
**SV1**														5.100	7.148	7.155	7.716	7.267	7.297	7.260	7.337	
**DN127**															7.423	7.430	7.986	7.621	7.568	7.539	7.621	
**ACA-1**																0.231	5.347	6.396	6.464	6.440	6.639	
**Pko**																	5.350	6.401	6.470	6.444	6.631	
**A14S**																		7.096	7.116	7.095	7.252	
**PBi**																			2.240	2.207	6.758	
**Far04**																				0.800	6.739	
**PBr**																					6.707	

^a^ Table values are percent differences in nucleotide sequence as determined from pairwise comparisons of the Mugsy alignment described in the Methods section.

The chromosomes of *B*. *burgdorferi* isolate B31, one *B*. “*bavariensis*” isolate (strain PBi), and one *B*. *afzelii* (PKo) isolate species have previously been reported to have nearly identical gene content [14,54]. We confirm these observations and extend them to additional species. Our data show that the sequences of *B*. *burgdorferi*, *B*. *afzelii*, *B*. *garinii* and *B*. *valaisiana* chromosomes are from 6.5% to 8.0% different from one another in between-species comparisons (Table 2). The chromosomes of *B*. *bissettii*, *B*. *spielmanii* and *B*. “*bavariensis*” (isolate PBi) are 5.0% different from *B*. *burgdorferi*, 5.3% different from *B*. *afzelii* and 2.2% different from *B*. *garinii*, respectively. The chromosome of the Finland isolate SV1 is 1.75 ± 0.3% different from the *B*. *burgdorferi* s.s. isolates (Table 2), and its plasmids are quite different from those of *B*. *burgdorferi* (our unpublished analysis). Thus, if as has been suggested by Margos and colleagues [48] isolate PBi represents a newly defined species *B*. “*bavariensis*”, then isolate SV1 might also be considered as representative of another previously undefined species, for which we have suggested the name *B*. “*finlandensis*” [53]. The observed differences between isolates *within* species, 0.35±0.27%, 0.23% and 0.80% for *B*. *burgdorferi s*.*s*., *B*. *afzelii* and *B*. *garinii*, respectively, are all considerably less than inter-species values and so robustly confirm these existing groupings (Table 2).

We have not performed an exhaustive comparison of the indels that relate the constant region of the *B*. *burgdorferi* s.s. chromosome to those of the other *B*. *burgdorferi* s.l. species, but nearly all of the *B*. *burgdorferi* chromosomal genes are present in chromosomes of each of the s.l. species. For example, our comparison of *B*. *burgdorferi* and *B*. *afzelii* shows that in addition to inter-species differences in the three repeat-containing genes mentioned above, we find only eleven indels larger than 25 bp that differentiate the chromosomes of the two species (Additional file 3: Table S3). Compared to strain B31, these include the previously described duplications in the *bmp* gene region in strains PBi and PKo [54,63], the differential presence of two genes (*0138* and *0223*), five indels <330 bp long between genes, and indels of <150 bp in genes *0309*, *0704* and *0749*. In addition, both *B*. *afzelii* isolates PKo and ACA-1 have an apparent duplication of the 16S rRNA gene region relative to other *B*. *burgdorferi* s.l. isolates. We note that the above non-VNTR indels are the same in the chromosomes of both sequenced *B*. *afzelii* genomes (Additional file 3: Table S3), again indicating the very similar nature of chromosomes within each species. Finally, an unusually variable region of the chromosome was identified in regions orthologous to B31 gene *b31*_*0524* (Additional file 4: Figure S1), which has suffered various deletions/insertions in the different species. It is possible that these chromosomal indel differences could be used for species identification.

The linear chromosomes of four members of the relapsing fever *Borrelia* clade have also been sequenced, those of *B*. *recurrentis*, *B*. *duttonii*, *B*. *hermsii* and *B*. *turicatae* ([64]; S. Porcella *et al*., unpublished Genbank accession nos. CP000048 and CP000049). In regions of homology, their chromosomes are typically about 20% different in sequence from the Lyme agent species, and form three subgroups: *recurrentis*/*duttonii*, *hermsii* and *turicatae* within which *hermsii* and *turicatae* chromosomes are 5-10% different and they in turn are 10-15% different from *recurrentis* and *duttonii* (from our analysis of a number of randomly chosen sequences scattered across the chromosomes; see also [46,65]). The relapsing fever clade chromosomes are generally syntenic with the Lyme agent chromosomes, but *B*. *recurrentis* and *B*. *duttonii* have about thirty gene content differences in the chromosomal “constant region” [64] (such an analysis has not been reported for *B*. *hermsii* and *B*. *turicatae*). It is clear that all known *Borrelias* have quite similar chromosomes.

### *Borrelia* plasmid diversity

Among the 14 *B*. *burgdorferi* s.s. isolates analyzed, the number of plasmids carried by each strain varies between 12 (strain CA-11.2A) and 21 (strains B31 and WI91-23) (Table 1). The other *B*. *burgdorferi* s.l. species carry on average somewhat fewer plasmids, between 7 in *B*. *garinii* Far04 and 17 plasmids in *B*. *afzelii* PKo (Table 1). We have previously argued that plasmid proteins encoded by paralogous family (PFam) 32 correlate with the compatibility type of *Borrelia* plasmids that are >10 kbp in length [57,59,63]. Our preliminary unpublished analysis suggests that the sequenced Lyme agent plasmids represent 29 PFam32 protein compatibility types, and probably several additional types that do not have PFam32 genes. Since B31 carries 19 different PFam32 type plasmids, and only ten “new” types are present in the 21 subsequently sequenced genomes, it seems likely that if other plasmid compatibility types remain to be discovered, they are not common. Analysis of all 23 Lyme agent genomes shows that plasmids cp26, lp54 and at least a few cp32s are always present and largely structurally conserved (with the single exception of *B*. *garinii* strain Far04 which has no cp32 plasmid). Some plasmids, lp5 and lp21, are less common and present in fewer than 10% of the analyzed isolates, while other plasmids are nearly always present but are organizationally variable (lp17, lp25, lp28-1, -2, -3, -4, -5, -6, -7, -8 and −9, lp36, lp38, lp56). We recently described an in-depth analysis of the plasmids present in four *B*. *burgdorferi* s.s. isolates [57,59,63], and a similar analysis of the additional plasmids sequenced in this study will be presented in a subsequent publication. Only the most highly conserved plasmids, cp26 and lp54, will be discussed further here.

### Single nucleotide polymorphism (SNP) analysis

To further resolve the population structure of *Borrelia* species and gain insights into the evolutionary history, gene sequence conservation and diversity across *Borrelia* isolates, we applied a single nucleotide polymorphism (SNP)-based genotyping methodology on the predicted gene sequences that are conserved across all sequenced *Borrelia* linear chromosomes and the lp54 and cp26 plasmids, using the *B*. *burgdorferi* B31 genome as reference. Only intragenic SNPs were considered, because the goal was to gain insights into protein sequence variations across the different isolates (Table 1). The small number of SNPs that might be present in the gaps between contigs in the chromosomes that remain in “draft” state would not be included in our analysis, but this does not affect any of the conclusions drawn below.

**Figure 2:**
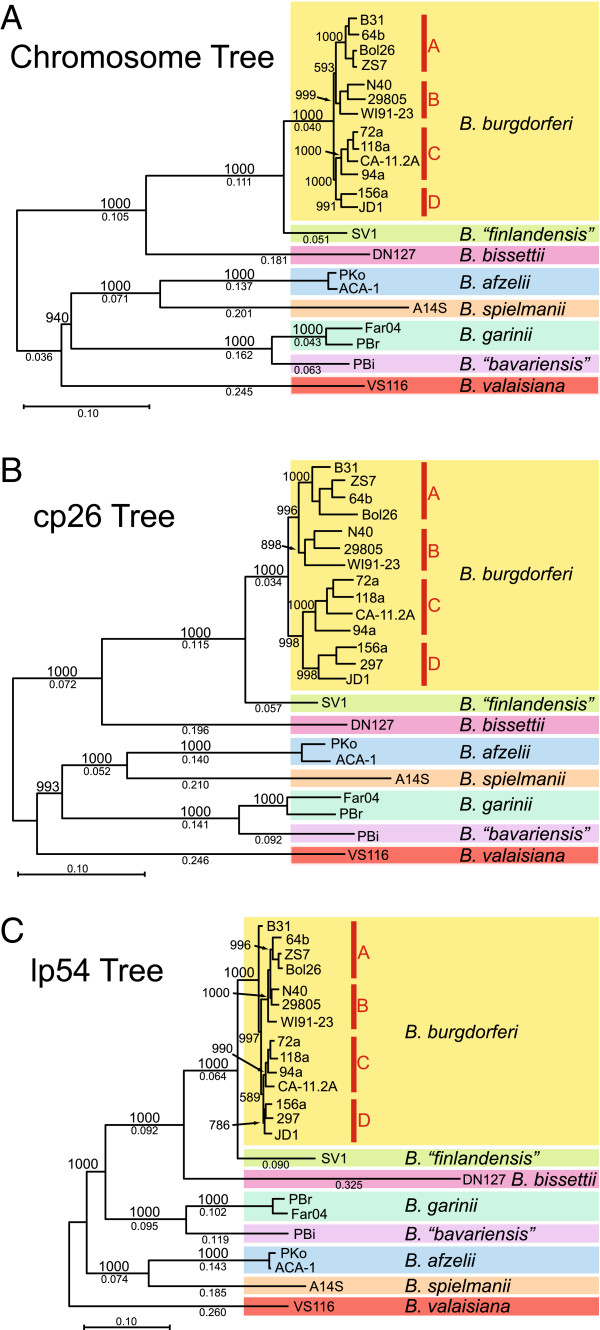
**Inter- and intra-species phylogenetic SNP trees for *B. burgdorferi *s.l.** Maximum likelihood SNP trees were constructed as described in the Methods section, using the strains are described in Table 1. Well-supported bootstrap values for 1000 trials are shown above the branches (values for short branches are not shown), and lengths are given below the branches. In each panel a scale of fractional difference is shown. The four *B*. *burgdorferi* s.s. groups are indicated in red (see text).

We identified a total of 10,299 synonymous SNPs (sSNPs) and 7,007 non-synonymous SNPs (nsSNPs) in cp26, 14,703 sSNPs and 13,514 nsSNPs in lp54, and 342,892 sSNPs and 178,324 nsSNPs in the chromosomes of the 22 isolates [66]. The SNP locations were concatenated into one sequence and a maximum-likelihood phylogenetic tree was built with this information (Figure 2). The branching orders of the different species are identical in the chromosome and cp26 SNP trees, indicating that these two replicons *as a whole* have not been reassorted during the evolution of the strains in this study. All three SNP trees are consistent with the previously delineated *B*. *burgdorferi* s.l. species, since the different species reside on strongly-supported, well-separated branches. All three trees show that *B*. “*finlandensis*” (isolate SV1) is a rather close relative of *B*. *burgdorferi* (Figure 2). Nonetheless SV1 is consistently and robustly separated from the *B*. *burgdorferi* s.s. strain cluster as well as from the other species, confirming the uniqueness of the SV1 isolate and its status as a potential new species [53]. The trees also show that *B*. *bissettii* strain DN127 is most closely related to *B*. *burgdorferi* s.s., *B*. “*bavariensis*” PBi is most closely related to *B*. *garinii*, and *B*. *spielmanii* A14S is most closely related to *B*. *afzelii*. *B*. *valaisiana* VS116 not particularly closely related to any of the other species in this study. The lp54 SNP tree is quite similar to the chromosome and cp26 trees (Figure 2C), except that the *B*. *garinii*-*B*. “*bavariensis*” cluster occupies a somewhat different position in the tree that is less closely associated with the *B*. *afzelii*-*B*. *spielmanii* branch than in the other two trees, and the *B*. *bissettii* lp54 plasmid is more divergent than its chromosome or cp26. The positions of the B31 and WI91-23 lp54s are also somewhat different within the *B*. *burgdorferi* s.s. cluster than in the other two trees. lp54, while not appearing to have diverged more rapidly than the chromosome or cp26, may have undergone horizontal exchange at more locations than the other two replicons [66].

This SNP-based phylogeny reveals a tight grouping of all the *B*. *burgdorferi* s.s. strains, a finding consistent with their overall sequence similarity (above) and previous analyses using rRNA IGS sequences [35,37] or protein-coding gene sequences in MLST analyses (Figure 1 and [33]). In addition, the chromosome and cp26 SNP trees (Figure 2A and B) give very similar well-resolved pictures of the phylogenetic organization of the *B*. *burgdorferi* s.s. clade. The three SNP-based trees have highly supported branch points and consistently identify four subgroups: strains B31/64b/Bol26/ZS7, strains N40/29805/WI91-23, strains 118a/72a/CA11.2A-94a, and strains 156a/297/JD1. These four subgroups are named SNP groups A, B, C and D, respectively, in Figure 2. Intra-species chromosomal diversity is about the same in the three cases where multiple genome sequences are available from a single species; *i*.*e*., the 12 chromosomes of *B*. *burgdorferi* isolates are about as divergent from one another as the two *B*. *garinii* and the two *B*. *afzelii* isolates are from each other (see also Figure 2).

Are these four SNP-based *B*. *burgdorferi* s.s. chromosome and cp26 SNP subgroups consistent with other chromosomal typing methods? The previous chromosomal typing strategies to which we can compare our genome sequence data are rRNA IGS1 sequence type [35,37], rRNA IGS1 restriction site type (RST) [67,68] and two MLST schemes: the MLST analysis of Margos *et al*. [33] (as applied by Travinsky *et al*. [38]) and our MLST analysis (Figure 1). Both MLST studies included information from a number of chromosomal genes and so incorporate more information than the IGS1 categorizations; they utilized different sets of eight and six chromosomal genes, respectively. Table 3 shows a compilation of the chromosomal “types” determined by these different methods, and it shows that SNP group A is convincingly supported by all four of the above analyses. These four strains clearly represent a separate chromosomal clade from the rest of the *B*. *burgdorferi* s.s. isolates. IGS1 and MLST analyses indicate that IGS1 types 1 and 3 form one major chromosomal clade and all the other types form a second major division (Figure 1 and [35]). Dissection of the second major IGS1/MLST division into convincing sub-clades has been less certain. The inclusion of strains 156a and 297 in SNP group D is in agreement with rRNA and MLST analyses that also show these two strains to be closely related, however only the SNP analysis includes JD1 in this cluster. SNP groups B and C together correspond largely to RST type 3, and some of these strains are similarly clustered by the other methods (*e*.*g*., IGS1 groups 94a, 118a and CA-11.2A together; the Travinsky *et al*. [38] MLST groups 72a, 118a and CA-11.2A together; and our MLST groups 94a and 118a together). However, there are also significant differences among the different grouping methods. For example, both MLST analyses place 118a and WI91-23 in the same group, while they robustly reside in SNP groups C and B, respectively; and JD1 is placed in a different group in each of these three analyses. Thus, although all three SNP, IGS1 and MLST typing methods display the genetic diversity of *B*. *burgdorferi* species, significant differences exist among these analyses, even when the two MLST analyses are compared. These differences are most likely due to the relatively small number of polymorphic sites at IGS1 and MLST loci, which subject the latter trees more strongly to the homoplastic effects of recombination.

**Table 3:** ***B*. *burgdorferi *** **chromosomal groups**

**Typing Method**^**a**^	1	2	3	4	5	6	7	8
**Strain**								
**B31**	1	1	1	1	1	1	1/2	A
**64b**	3^b^	1	1	1	1	1	1/2	A
**Bol26**	1	–	3	1	1	1	1/2	A
**ZS7**	1	1	3	1	1	1	1/2	A
**156a**	2	2	2	2	2	2a	2/1	D
**297**	2	2	–	2	2	2b	–	D^c^
**JD1**	5	1	6	3	2	2c	2/1	D
**72a**	4	2	4	3	3	3a	1/1	C
**94a**	8^b^	3	4	3	3	3a	1/1	C
**118a**	5	2	4	3	3	3b	1/1	C
**CA-11.2A**	5	2	2	3	3	3a	1/–	C
**N40**	9	3	5	3	4	4	2/2	B
**WI91-23**	7	2	4	3	5	4	2/1	B
**29805**	6	3	7	3	6	4	1/1	B

a. Chromosomal typing methods are as follows:

1. Ribosomal r*RNA* spacer *IGS*1 [35,37].

2. Arbitrary *MLST* group name [33,38].

3. Arbitrary *MLST* group name (Figure 1 this report).

4. RST type deduced from our sequences [67,68].

5. Plasmid content similarity, arbitrary group name (S. Casjens, unpublished).

6. Chromosomal right end organization, arbitrary group name (S. Casjens, unpublished).

7. Two chromosomal indels: 157 bp in gene *0021* and 27–30 bp between genes *0001* and *0002* (1 = no deletion; 2= deletion).

8. Chromosomal, cp26 and lp54 *SNP* type, this report.

- not determined.

b. *IGS*1 lineages 1 and 3 form a coherent superlineage, and *IGS*1 8 is a rather close relative of lineage *IGS*1 5 [35].

c. 297 chromosomal *SNP* type not determined.

In Table 3 we also show a summary of the following three additional categorizations which are not subject to homoplasy issues: (i) Linear plasmid contents indicate that these are most similar within the following three groups: B31/64b/Bol26/ZS7, 156a/297/JD1, and 118a/72a/CA11.2A/94a (our analysis to be published elsewhere) which correspond perfectly to SNP groups A, D and C, respectively; (ii) the plasmid accretion events at the chromosome’s right end are fully consistent with the four SNP groups (our analysis to be published elsewhere); and (iii) the 157 bp deletion in chromosomal gene *b31*_*0021* that is present in some *B*. *burgdorferi* s.s. strains (Additional file 3: Table S3) is limited to SNP groups B and D (both B type genomes have the deletion and two of the three group D chromosomes carry it). The fact that B type strain 29805 does not have the deletion, along with the facts that the linear plasmids of the three type B isolates are not particularly similar to each other or the other isolates and that the D strains are not clustered by IGS1 or MLST methods (the only exception to the latter is that N40 and 29805 are closely related to each other in the Travinsky *et al*. [38] MLST analysis), suggest that SNP group B strains may be less uniform than the other three SNP groups. The overall agreement between SNP analysis and the plasmid content, chromosome right end structure and deletions in homologues of *gene 0021*, along with the extremely large number of alleles in the SNP analysis, lead us to conclude that the SNP groups most accurately reflect the real average evolutionary history of the chromosome. Of course, occasional horizontal exchange of approximately gene sized DNA fragments is known to happen and can lead to differences in the evolutionary histories of any given locus [66].

### The *Borrelia* pan-genome

The genetic repertoire of a given species can be much larger than the gene content of individual strains, as the gene content of individual strains of the same species can vary considerably, and new genes continue to be discovered even after sequencing the genomes of many isolates. This observation has led Tettelin and colleagues to introduce the concept of “pan-genome”, defined as the sum of the core genome (genes shared by all strains) and the dispensable genome (genes absent from one or more strains, and genes that are unique to each strain) [69-71]. The core genome typically comprises the genes that encode essential functions related to the basic biology of the species, whereas the dispensable genome contributes to species’ diversity and provides functions that are not essential to its basic lifestyle but that may confer selective advantages (niche adaptation, antibiotic resistance, ability to colonize new hosts, *etc*.). In order to understand the basic biology and population genetics of any species, the core and dispensable genomes must be known.

**Figure 3:**
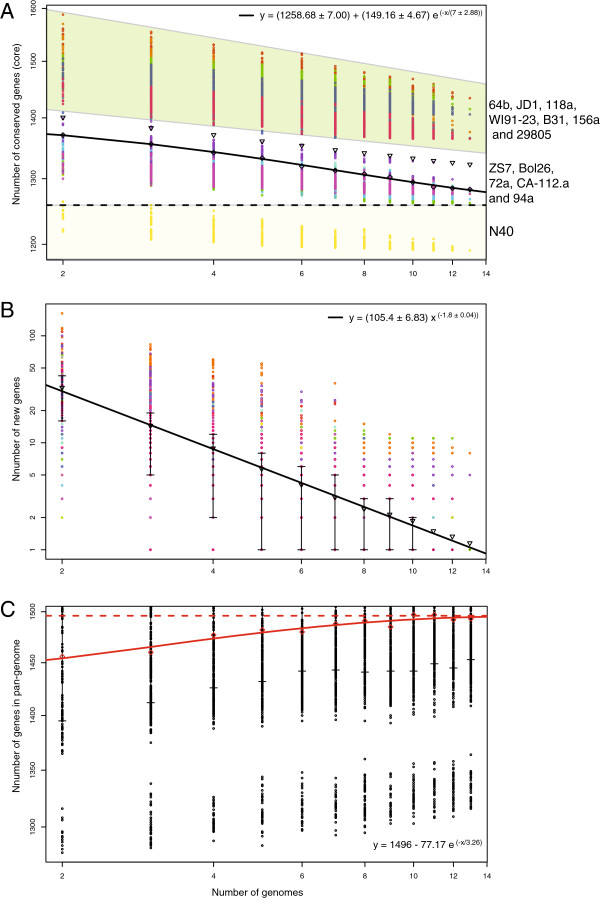
**Pan**-**genome calculations of the conserved core, predicted new genes and pan**-**genome size of thirteen *B. burgdorferi *s**.**s**. **isolates. (A)** Conserved genes (core genome) plotted as a function of the number *n* of sequenced genomes (x-axis). For each *n*, colored data point represents values of the number of conserved genes obtained for all possible combinations of compared genomes. The black line shows the exponential decay model based on the median value for conserved genes when increasing numbers of genomes are compared. **(B)** Plot showing decreasing numbers of discovered new genes with increasing number of genomes compared. The black line shows the exponential decay model based on the median value for new genes when increasing numbers of genomes are compared. **(C)** Pan-genome of the *B*. *burgdorferi* species. The extrapolated curve plateaus at a value of about 1500 with 13 genome sequences, and as a consequence, *B*. *burgdorferi* s.s. has a closed pan-genome.

**Figure 4:**
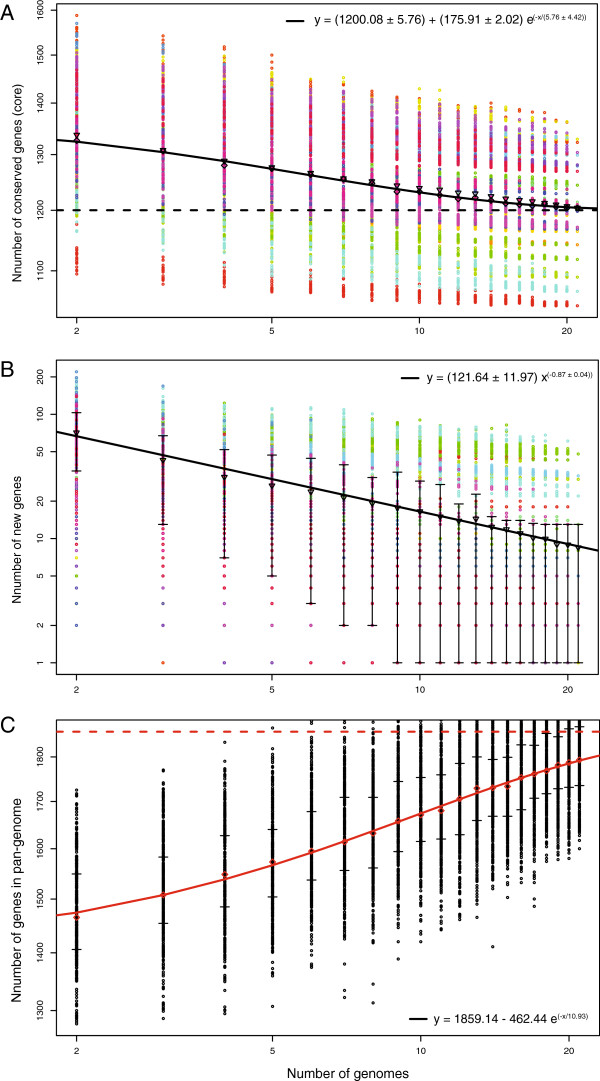
**Pan-genome calculations of the conserved core**, **predicted new genes and pan**-**genome size of 21 *B. burgdorferi *s.s. and s.l. isolates.** The pan-genome calculations displayed in the figure were performed in order to gain insight about the entire genomic diversity of the *B*. *burgdorferi* s.l. group. **(A)**, Each colored data point represents values of the number of conserved genes calculated for different combinations of *n* compared genomes (*n* value plotted on the x-axis). The exponential decay model (black solid line), based on the median value for conserved genes when increasing numbers of genomes are compared, predicts a conserved gene core for *Borrelia* sp. of 1200 genes. **(B)** Plot showing decreasing numbers of predicted new genes with increasing number of genomes compared. The black line shows the exponential decay model based on the median value for new genes when increasing numbers of genomes were compared. **(C)** The extrapolated curve plateaus at a value of 1859 genes, that is reached for a total of 21 genomes, highlighting the fact that there is some genomic diversity left to be discovered in *B*. *burgdorferi* s.l. isolates.

One approach to estimate the extent of the core and dispensable genome components is to compute the number of new genes identified each time a new genome of a species is sequenced. Each of the core and dispensable genomes should approach their true values as more genomes are analyzed, assuming the isolates chosen for sequencing span the genomic diversity of the species. This strategy was applied here for *B*. *burgdorferi* s.s. isolates (Figure 3), as well as *B*. *burgdorferi* s.l. isolates (Figure 4). Pan-genome calculations have not been previously applied to *Borrelia* due to the lack of high-quality complete genome sequence information.

#### The *B. burgdorferi* sensu stricto pan-genome

Comparative genome analysis performed with 13 *B*. *burgdorferi* s.s. genomes (Table 1) provides a picture of the genetic diversity within this species. Extrapolation of the exponential decay model shown in Figure 3A suggests that the size of the conserved gene core for the *B*. *burgdorferi* species reaches an asymptote with the comparison of ~20 *B*. *burgdorferi* genomes, for a number of core genes of ~1250 genes (dashed line in Figure 3A). The model is based on the median number of conserved genes in each of the permutations of all possible genome comparisons. The functions encoded by the conserved core genome closely follow the distribution profile of the functional categories encoded in the entire *Borrelia* genome; *e*.*g*. there is no enrichment for a particular functional category in the set of conserved core genes. A total of 30.9% of the conserved core genes are hypothetical proteins (no sequence similarity outside of *Borrelia* and no predicted protein domains and motifs), highlighting the potentially important roles of these unknown genes in the *Borrelia* physiology.

Closer examination of the conserved core genes plot reveals 3 clusters of data points (Figure 3A). The first group of points, the yellow circles below the line y=1258 (Figure 3A), represent the genome comparisons in which the N40 strain is used as the reference. The size of the N40 core genome, compared to the other *B*. *burgdorferi* isolates, is approximately 1194 genes. The data points highlighted by the green area in Figure 3A, represent the genome comparisons where 7 strains are used as reference in permutations of genome comparisons: B31, 118a, 156a, 29805, 64b, JD1 and WI91-23. This group of strains is characterized by a larger core genome (>1350 genes). Finally, the third group of points is represented by the following 5 isolates: ZS7, Bol26, 72a, CA-112.a and 94a (1350 genes > core genome size > 1194 genes).

A likely explanation for the observation that permutations in which N40 is used as the reference genome lead to low values of predicted conserved gene core (Figure 3A) is that the N40 genome has a lower gene redundancy when compared to the other *B*. *burgdorferi* genomes. In order to test this hypothesis, we performed a Jaccard Orthologous Clustering (JOC) analysis on 21 *Borrelia* s.l. genomes. JOC analysis is typically used to group together highly similar proteins within a single genome/organism of interest and allows for 1-to-many orthology. JOC analysis has been applied to the analysis of various organisms such as *Streptococcus pneumoniae*[72], *Neisseria meningitidis*[73] and *Plasmodium*[72]. The Jaccard clustering analysis predicted a total of 1,479 orthologous protein clusters, each containing between 1 to 239 proteins (all genomes combined) (Additional file 5: Table S4). The protein cluster with the largest number of members, 239 proteins across all the genomes, has no known function and is encoded on the cp32 plasmids (a representative protein is *b31*_*l02* which has been speculated to be a virion morphogenesis gene on the cp32 prophage plasmids [57,74,75]). On average, for the 100 orthologous protein clusters with the most members (representing a total of 7930 proteins across the 22 genomes included in the analysis), N40 had clusters with 26.2% fewer members than the equivalent clusters in strain B31 (Additional file 5: Table S4). This result confirms that the N40 genome has the lowest gene redundancy, in part contributing to the N40 smaller gene core.

Our pan-genome analysis also estimated the size of the dispensable genome of *B*. *burgdorferi* s.s., *i*.*e*. the genes absent from one or more strains and the genes that are unique to each strain (Figure 3B). The large deviation from the mean shown in Figure 3B, ranging from only a few to over one hundred genes, is a reflection of the genetic variation within *B*. *burgdorferi*. The regression model in Figure 3B shows a rapid decrease in the predicted number of new genes discovered for each new *B*. *burgdorferi* genome sequenced. This number reaches 0 for a number of genomes close to 14 (regression crossing the X-axis in Figure 3B), confirming that the 13 *B*. *burgdorferi* genomes included in our study should nearly cover the complete genetic diversity of the species and that the sequencing of additional strains (unless they came from a currently undiscovered clade) would add only marginally to the known genetic pool.

The pan-genome plot in Figure 3C represents an estimation of the complete *Borrelia* s.s. gene pool based on the set of genomes analyzed. The extrapolated curve ceases to increase as new genomes are added to the analysis and reaches a plateau of approximately 1500 genes: thus, the *B*. *burgdorferi* s.s. pan-genome is a “closed” pan-genome.

#### The pan-genome of *B. burgdorferi* sensu lato species

Because of their conserved synteny and highly related sequences, we also applied the pan-genome analysis to the *B*. *burgdorferi* s.l. group of species, including *B*. *valaisiana*, *B*. *afzelii*, *B*. *garinii*, *B*. *bissettii* and *B*. *spielmanii*. Calculation of the conserved core genome size predicted approximately 1200 core genes across 21 *B*. *burgdorferi* s.l. genomes (Figure 4A). The exponential decay shown in Figure 4A is based on the median number of genes for each genome comparison, and reaches an asymptote with the comparison of about 20 genomes. The range of size of the core genomes is broad, and varies depending on the combination of genomes being compared, from 1,046 genes for strain Far04 (red dots at the bottom of the plot in Figure 4A) to 1,329 genes for strain JD1. The strains with the largest core genomes are JD1 (1,329 genes), 64b (1,323 genes), and 118a (1,304 genes). The strains with the smallest core genomes are Far04 (1,046 genes), PBr (1,068 genes), A14S (1,090 genes) and N40 (1,142 genes). Examination of the protein clusters from the JOC analysis (Additional file 5: Table S4) showed that only 1,003 orthologous protein clusters were identified in the genome of strain Far04 (the smallest of the genomes analyzed here), compared to a total of 1,081 for strain B31. The lower number of protein clusters predicted for stain Far04, reflecting the absence of proteins otherwise present in the other *Borrelia* s.l. genomes, is the main contributing factor explaining the Far04 smaller conserved core.

With 21 genomes compared, the predicted median number of new genes discovered per genome sequenced is 12 genes (Figure 4B). This suggests that there is still some genomic diversity left undiscovered *within* the *Borrelia* s.l. species although it may not be great. However, this predicted number of new genes is not homogenous and varies quite significantly depending on the genome considered, as highlighted by the wide variability in the data points in Figure 4B. The pan-genome analysis identified 48 unique genes in VS116, 52 for strain DN127, 30 for strains A14S and 22 for strain PBr. In comparison, no unique genes were predicted for strains 64b, 118a, B31, 156a, Bol26, ZS7, 72a, CA-11.2a and N40 when compared to the other 20 genomes, confirming the results from the pan-genome analysis of the *B*. *burgdorferi* s.s. strains.

The pan-genome analyses we describe here might help predicting the genomic basis of human pathogenicity in *B*. *burgdorferi* s.l. For example, we identified genes uniquely present or absent in the genomes of *B*. *burgdorferi* s.s., *B*. *afzelii*, and *B*. *garinii*, the three species causing the majority of known cases of Lyme borreliosis. Three uncharacterized B31 plasmid genes (*e0040*, *d0031*, and *f06*) are present in all *B*. *burgdorferi* s.s. genomes while being absent in all other species including its closest outgroup SV1. The two *B*. *afzelii* genomes (PKo and ACA-1) have no uniquely present genes and one uniquely absent gene (*far04*_*0259*, uncharacterized). The two *B*. *garinii* genomes (PBr and Far04) contain 69 uniquely present genes, one of which is predicted to be an adenine-specific DNA methyltransferase (*far04*_*e0022*). Intriguingly, a large number of *Erp* (*ospE*-related protein) genes are missing in *B*. *garinii* genomes. The relatively large number of uniquely gained and lost genes may be a result of adaptation of *B*. *garinii* to its avian reservoir hosts [47]. Lineage-specific genes in these three highly pathogenic species, most of which are encoded in the plasmids, are listed in Additional file 6: Table S5. Each of the three species was represented by at least two genomes, further strengthening the predictions of presence or absence of specific genes in the different *Borrelia* lineages. While these genes are strong candidates contributing to human virulence, there are also possibilities of neutral gene acquisition or loss.

The *B*. *burgdorferi* s.l. pan-genome size calculation (Figure 4C) suggests that the pan-genome of this group is open, *e*.*g*. the extrapolated curve continues to increase as new genomes get added to the analysis.

### Phylogeny-based pan-genome analysis

**Figure 5:**
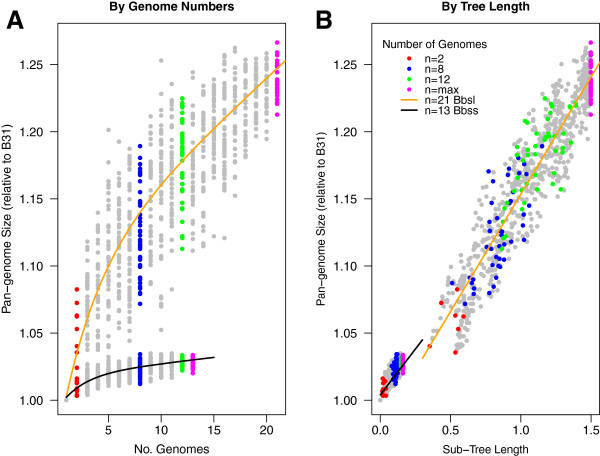
**Phylogenetic correlation of *B. burgdorferi *s.l. and *B. burgdorferi *s.s pan-genomes. (A)** Pan-genome sizes, calculated in permutations where the B31 genome is used as reference, are plotted against the number *n* of genomes in the comparisons. Data points were fitted with negative exponential growth models (see Methods) [69]. The least-square fitted model for *B*. *burgdorferi* s.l. (solid orange line) takes the following parameter values (and respective standard errors): D=0.995 (0.0121), *tg*(θ)=0.00725 (0.00083),κ=0.0445 (0.0187), and τ_*n*_=3.47 (1.06). The model fit has an R^2^=0.7479. The model for *B*. *burgdorferi* s.s. (solid black line) has the following parameter values (and respective standard errors): D=1.001 (0.00276), *tg*(θ)=0.000917 (0.00027),κ=0.01446 (0.0088), and τ_*n*_=*2*.*287* (*0*.*857*). The model fit has an R^2^=0.6065. **(B)** The same pan-genome calculations were fitted linearly with the sub-tree length of genomes in comparison, according to the neutral coalescence model: Ω_n_=Ω_0_+ωT_n_, where ω is the rate of gene acquisition [77]. The linear model for *B*. *burgdorferi* s.l. has Ω_0_=1458 (3.8) and ω=260.3 (3.5) with a greatly improved R^2^=0.8762 (p<2e-16). The linear model for *B*. *burgdorferi* s.s. has Ω_0_=1501 (0.91) and ω=202.3 (0.05) with a slightly improved R^2^=0.6126 (p<2e-16). The improvement is due to a tighter fit of predicted pan-genome sizes with the tree lengths, in which some groups of genomes are more phylogenetically diverse (thus having larger pan-genome sizes) than others albeit the total number of genomes in comparison remain the same (e.g. comparing n=8, in blue, in the two panels).

Traditional pan-genome analysis, as applied above, uses the number of genomes as the sole explanatory variable. In some cases, this approach insufficiently captures variations in the pan-genome because of the underlining phylogenetic structure among the genomes. Phylogenetic autocorrelation among individual strains is common even in bacterial species with moderate amount of recombination such as *E*. *coli* and *B*. *burgdorferi*[66,76]. Except in an idealized population where individual genomes are equally related to each other (*i*.*e*., a ”star phylogeny”), sampling an increasing number of genomes lead to an early plateau of the pan-genome size since random sampling quickly exhausts the total amount of phylogenetic diversity (Figure 5A, black line).

Pan-genome predictions could be improved by taking into account the phylogenetic relatedness of the genomes. A coalescence-based approach to pan-genome prediction has previously been applied to *Streptococcus pneumoniae*[77], and it has been shown that the linear dependence of pan-genome size on level of polymorphisms can be readily interpreted by the coalescence process [77]. In the present analysis, we used the length of the sub-tree connecting the genomes as a proxy for the total coalescence time among a set of genomes, an approach validated by the strong linearity of the relationships (Figure 5B). A molecular phylogenetic tree theoretically reflects gene coalescence history closely when there is no recombination between strains. Here, the level of recombination appears not high enough to distort genome phylogenies due to the fact that these trees are based on a large number of SNPs (Figure 2).

The coalescence model, and its extension to evolution between populations, requires a single explanatory parameter, the rate of gene acquisition *ω*. This model removes the phylogenetic autocorrelation in nonlinear models, and assumes that the bacterial pan-genome is mainly dependent on the total tree distance between the genomes compared. Thus, any new genome added to the analysis can theoretically increase the pan-genome size proportionally to its contribution to the total genome tree. In the coalescence model, there is therefore no concept of an ”open” or ”closed” pan-genome, as defined using previous strategies [71], but instead, the upper limit of a species pan-genome is solely determined by its overall phylogenetic diversity. Considering that the selection of bacterial isolates for genome sequencing is generally not random, the coalescence model might prove more robust in predicting bacterial pan-genomes. The coalescence model validates our genome sequencing strategy to sequence major evolutionary lineages within and between *B*. *burgdorferi* s.l. species, which maximizes pan-genome coverage for a given number of genomes.

Based on the coalescence model, expanding the set of genomes by sequencing additional *B*. *burgdorferi* s.s. lineages or *B*. *burgdorferi* s.l. species would extend the curves but not change the linear models themselves. According to the linear dependency of pan-genome sizes on the chromosomal SNP tree (Figure 2A), we estimate that the rates of gene acquisition within and between species are, respectively, 202.3 ± 0.05 and 260.3 ± 3.5 genes per unit tree distance, which is one substitution per nucleotide site on the main chromosome (Figure 5). The gene acquisition rate is thus 30% higher for between-species divergence than for within-species divergence. Since *B*. *burgdorferi* s.l. species may differ in vertebrate host preference but not necessarily in their tick vectors, one contributing factor to the accelerated gene acquisition between species could be host adaptation.

Considering the genetic distances (Table 2) for a pair of *B*. *burgdorferi* s.s. strains – about 0.50% per nucleotide site and a pair of *B*. *burgdorferi* s.l. species - 7.0%, the above gene-acquisition rates translate to a gain of roughly 1.0 (=202.3 X 0.005) novel gene during strain divergence within species and 18 (=260.3 X 0.07) genes between species. Based on the length of the aligned common sequences of the main chromosomes (L=843,710 bases), these gene-acquisition rates translate to 2.4X10^-4^ (=202.3/L) SNPs within *B*. *burgdorferi* s.s. and 3.1x10^-4^ (=260.3/L) genes per SNP between *B*. *burgdorferi* s.l. Both rates are two orders of magnitude lower than the reported gene acquisition rate of 0.017 ± 0.002 genes per SNP in *Streptococcus pneumonia*[78]. These low gene-acquisition rates suggest that our previous conclusion of the gene-content stability of *B*. *burgdorferi* s.s. holds true for the entire *B*. *burgdorferi* s.l. species group [55]. Indeed, *B*. *burgdorferi* s.l. appears to have one of the largest core genomes among bacterial pathogens, which amounts to 83.9% (=1258.58/1500) of the pan-genome within *B*. *burgdorferi* s.s. (Figure 3) and 64.6% (=1200/1859) of the pan-genome *B*. *burgdorferi* s.l. (Figure 4). In comparison, the proportion of the core genome relative to the pan-genome ranges from 8.6% in *Clostridium botulinum* to 41.1% in *Yersinia pestis* by one survey [78] and from 44% in *Escherichia coli* to 98% in *Mycobacterium tuberculosis* by a more recent account [79]. Genome stability in *B*. *burgdorferi* s.l. and others may be a reflection of low rates of horizontal gene transfer and, ultimately, narrow ecological niches of these bacterial species [79]. Based on the high degree of genome stability of *B*. *burgdorferi* s.l. and the large variations in the size of paralogous gene families among strains (Additional file 5: Table S4 and Additional file 6: Table S5), we conclude that adaptive genome evolution in *B*. *burgdorferi* s.l. is driven primarily by duplication and loss of genes (especially lipoprotein genes) and not by acquisition of new genes through horizontal gene transfer. Nevertheless, variations in gene regulatory sequences may contribute to the adaptive genome divergence in *B*. *burgdorferi* s.l. as well.

**Figure 6:**
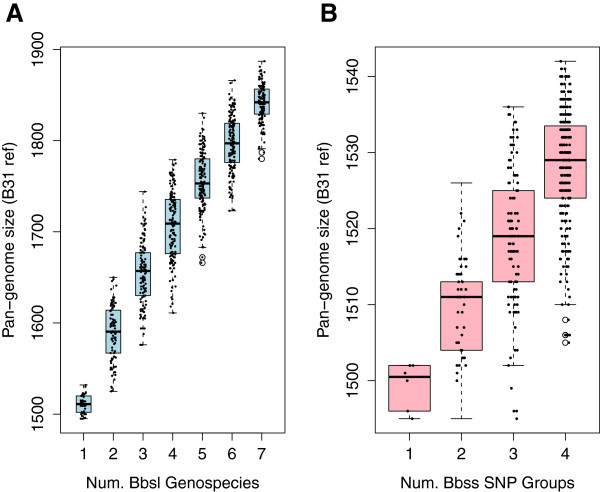
**Phylogenetic prediction of future pan-genome growth.** Using the same dataset as in Figure 4, the pan-genome sizes (*y*-axis) were re-plotted against the number of distinct phylogenetic groups (*x*-axis) these genomes represent, another phylogeny-guided way to linearize the prediction of pan-genome sizes. **(A)** Each point represents the pan-genome size (*y*-axis) of a group of randomly selected genomes. The number of distinct *B*. *burgdorferi* s.l. species these genomes represent is plotted on *x*-axis. Based on the linearity of the median values, we estimate that sequencing an additional species would yield ~100 new genes. **(B)** Each point represents the pan-genome size (*y*-axis) of a group of randomly selected genomes. The number of distinct *B*. *burgdorferi* s.s. SNP groups these genomes represent is plotted on *x*-axis. Predicted increase of the *B*. *burgdorferi* s.s. pan-genome with increasing number of SNP groups. Based on the linear median values, we estimate that sequencing an additional new SNP group (e.g., a European lineage) would yield merely ~10 new genes.

The phylogeny-based pan-genome models allow for a more accurate prediction of the pan-genome size, providing a robust guidance for future genome sequencing efforts in *B*. *burgdorferi* s.l. In Figure 6, the predicted pan-genome sizes, calculated from genome permutations in which B31 was used as the reference, are plotted against the total number of phylogenetic groups. The goal of this approach was to determine how the predicted *B*. *burgdorferi* s.l. pan-genome size might be affected by sequencing additional phylogenetic groups. The addition of each species genome to *B*. *burgdorferi* s.l. increases the pan-genome size by approximately 50–100 distinct genes (3–7% of a 1500-gene genome; Figure 6A). Since the overall relationship is linear (Figure 6A), we predict based on this model that future sequencing of new *B*. *burgdorferi* s.l. species is likely to add similar numbers of new genes. By the same reasoning, this analysis predicts that sequencing an additional *ospC*-typed genomic group (including those from Europe; see Figure 1) would add little (~10 new genes, <1% of a genome) to the pan-genome of *B*. *burgdorferi* s.s. (Figure 6B).

### ORF sequence variation in *Borrelia* spp

To quantify evolutionary constraints on the amino acid variations at each ORF locus, we calculated maximum likelihood estimates of synonymous (*K*_*S*_) and nonsynonymous (*K*_*A*_) nucleotide substitution rates between B31 and the other s.s. and s.l. strains by using the PAML package [80] (individual values of *K*_*A*_, *K*_*S*_, *and K*_*A*_/*K*_*S*_ in Additional file 7: Table S6). Among the three replicons, ORFs on cp26 have the highest average *K*_*S*_ values (*p*=*10*^-*6*^ in a *t*-test of within-species *K*_*S*_ values between cp26 and lp54; Table 4). The elevated *K*_*S*_ values on cp26 are almost certainly a result of selectively maintained high localized recombination rates in regions surrounding *ospC*[66]. ORFs on lp54 show significantly higher *K*_*A*_/*K*_*S*_ ratios than those on cp26 and the main chromosome for both the within- and between-species comparisons (*p*=*10*^-*9*^ in a *t*-test of within-species *K*_*A*_/*K*_*S*_ ratios between cp26 and lp54; Table 4). The relatively high *K*_*A*_/*K*_*S*_ ratios of ORFs on lp54 suggest a high level of adaptive amino-acid variations on this plasmid, an explanation consistent with the high proportion of genes encoding surface-localized lipoproteins on this plasmid including *ospA*, *ospB*, *dbpA*, *dbpB*, as well as the PFam54 array of CRASP-1-like genes [57,63,81]. On all three replicons, the within-species *K*_*A*_/*K*_*S*_ ratios are greater than the between-species *K*_*A*_/*K*_*S*_ ratios (Table 4), A higher level of amino-acid variations within- than between-species may be a result of slightly deleterious mutations within bacterial populations [82]. It is also possibly due to the fact that some amino-acid variations (such as those at *ospC*) are beneficial and selectively maintained within *B*. *burgdorferi* s.s. populations. Further investigation (e.g., by simulations) is needed to evaluate these possibilities.

**Table 4:** **Synonymous and non**-**synonymous SNPs**

	**cp26**	**lp54**	**Chromosome**
**No. of sSNPs**	10,299	14,703	342,892
**No. of nsSNPs**	7,007	13,514	178,324
**B31 vs**. **other *B. burgdorferi *** **sensu stricto strains**			
***K***_***A***_	0.002302^a^	0.001707^b^	0.001033
***K***_***S***_	0.01924^a^	0.006192^b^	0.007981
***K***_***A***_**/*K***_***S ***_**ratio**	0.120^a^	0.276^b^	0.129
**B31 vs. other *B. burgdorferi *** **sensu lato strains**			
***K***_***A***_	0.0243^a^	0.03530^b^	0.01403
***K***_***S***_	0.2907^a^	0.2309^b^	0.1573
***K***_***A***_**/*K***_***S ***_**ratio**	0.0837^a^	0.153^b^	0.0892

^a^ Mean value with *ospC* (*b19*) excluded

^b^ Mean value with *dbpA* (*a24*) excluded

**Figure 7:**
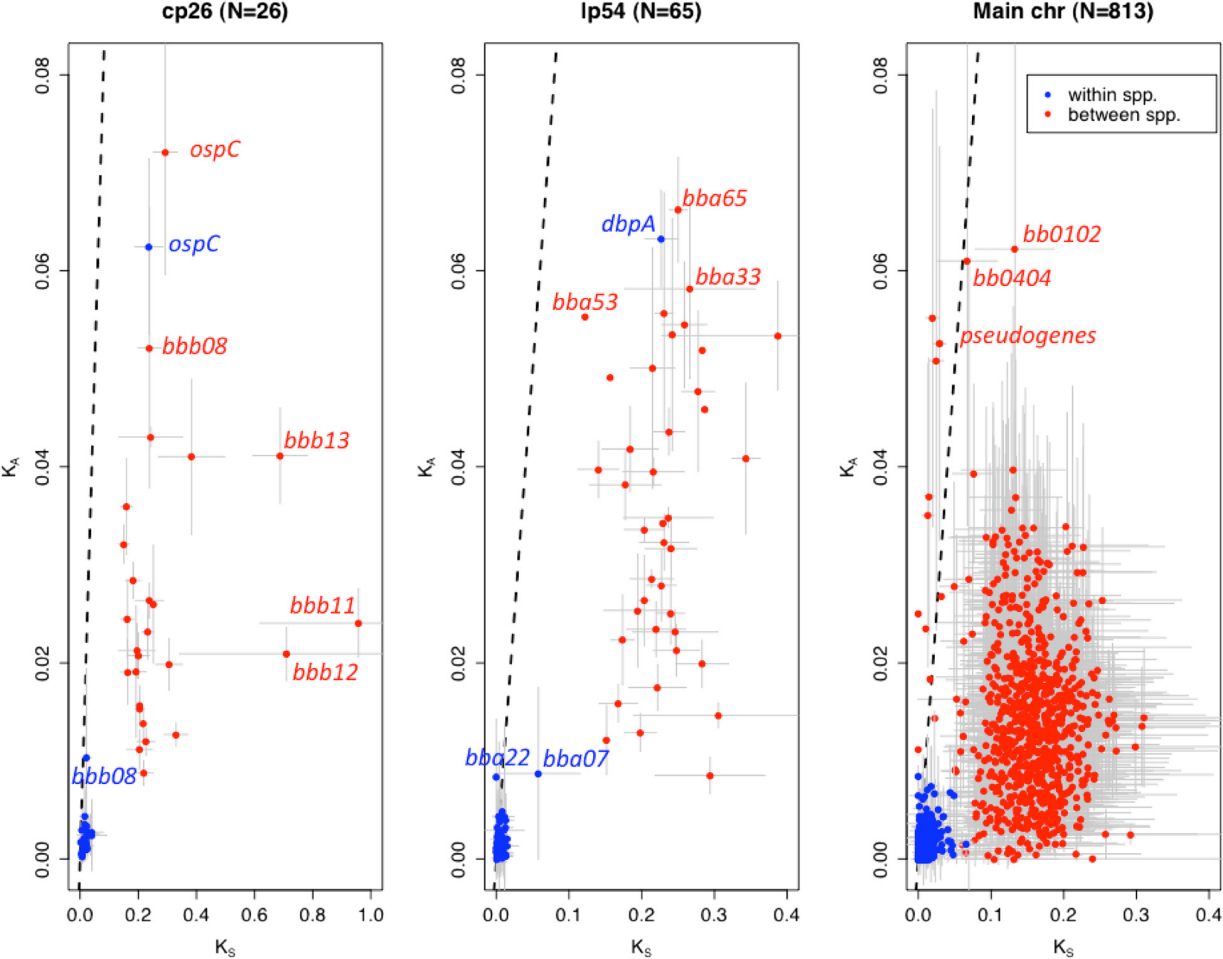
**Synonymous and nonsynonymous substitution rates of genes.** Each point represents the average synonymous (*K*_*S*_) and nonsynonymous (*K*_*A*_) rates between B31 and other strains for a given ORF locus. The average was obtained separately for comparisons of B31 with a strain of the same species (in blue) and with a strain of a different species (in red). Gray lines show one standard deviation of these within- and between-species comparisons. Within each panel, a dash line indicates the neutral expectation (*K*_*A*_ =*K*_*S*_).

Two genes, *ospC* and *dbpA*, were excluded from the above analysis because their *K*_*A*_ and *K*_*S*_ values were clearly outliers compared to other ORFs (Figure 7). These two genes are unique in having similar within- and between-species *K*_*A*_ and *K*_*S*_ values, a result consistent with the presence of strong balancing selection within *B*. *burgdorferi* populations [66]. These two genes are among the genes highly and exclusively expressed during host invasion [83-85]. To a much lesser extent the cp26 gene *b08*, which encodes a putative lipoprotein [86], shows unusually high *K*_*A*_ values in both within-species and between-species comparisons (Figure 7). Two genes on lp54, *a07* (putative ChpAI protein) and *a22* (hypothetical protein), showed high within-species *K*_*A*_ but normal *K*_*A*_ and *K*_*S*_ values in the between-species comparison. A large number of ORFs on lp54, most of which are predicted to encode lipoproteins, showed high *K*_*A*_ values in between-species comparisons, including *a65* (a CRASP-1 family gene), *a53* (function unknown), *a33* (encodes a putative lipoprotein) and *a54* (function unknown) (Figure 7). It should be noted that 29 ORFs on lp54 (that include ORFs encoding DbpA and CRASP-1) are missing in the between-species comparisons, because of the many gaps in the sequence alignments due to high sequence divergence. Two ORFs on the main chromosome (*0102* and *0404*, both of unknown function) also showed high *K*_*A*_ values in between-species comparisons. Since these genes have significantly high *K*_*A*_ values within populations, between populations, or both, their amino acid variations may be adaptive. In contrast, three genes involved in plasmid partitioning on cp26 (*b11*, *b12*, *and b13*) showed unusually low *K*_*A*_/*K*_*S*_ ratios, suggesting a high degree of amino-acid sequence conservation (Figure 7).

### Adaptive genome radiation as population expands

**Figure 8:**
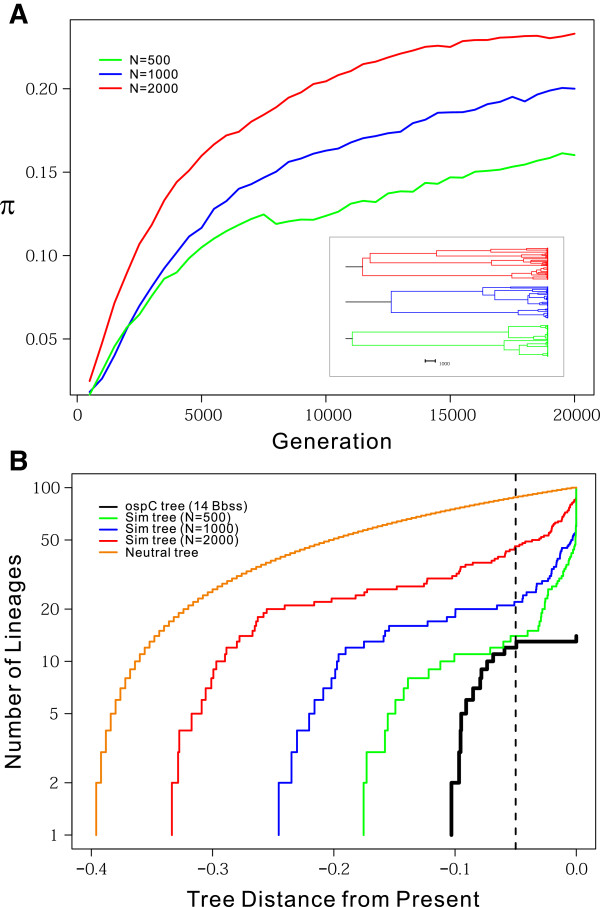
**Predicted increase of genomic diversity with *B***. ***burgdorferi *population expansion.** We simulated evolution of three bacterial populations, each of which consisted of a constant size of N=500 (in green), 1000 (in blue), or 2000 (in red) haploid genomes. One of the eight genes in the simulated genome evolves under immune-mediated negative frequency-dependent selection (FDS) as *ospC* is likely to be. Other genes were under purifying selection like housekeeping genes. **(A)** We measured nucleotide diversity at the FDS locus by sampling 100 individuals every 500 generations. Doubling of the population size results in a proportional increase in nucleotide diversity. (Inset) We reconstructed the coalescence trees for the last population samples. These trees show characteristics of balancing selection such as elongated internal branches and compressed terminal branches. **(B)** Lineage-Through-Time (LTT) plots of the tree based on *ospC* sequences from 14 *B*. *burgdorferi* s.s. genomes (in black), a simulated neutral coalescence tree (in orange), and trees of FDS sequences from simulated populations (in green, blue, and red). In comparison with the neutrally evolving genomes, the LTT plots of balanced gene trees show early rapid rise of gene lineages that are subsequently maintained for a long period of time, before the final rapid rise to the sample sizes.

While evolutionarily stable, as *B*. *burgdorferi* s.l. populations expand its genomes are expected to diversify rapidly through sequence and copy-number variations at host-interacting loci such as *ospC*. Mathematical analysis showed that the mean coalescence time of *n* segregating alleles at a locus under balancing selection is extended–relative to the neutral expectation–by a scaling factor: *E*{*T*_*n*_} = *2N*_*e*_*f*_*s*_(*1*-*1*/*n*) [EQ.1], where *N*_*e*_ is the effective population size and *f*_*s*_ is the scaling factor that increases with selection intensity [87]. The same analysis suggested rapid emergence of new alleles at such a locus as a population grows. For example, when selection is strong and the population size (after expansion) is large, the time for emergence of new alleles at such a locus in a diploid population is a small fraction of *2N* generations or, more precisely, in the order of *1*/(*4MS*) [EQ.2], where *M*=*N*_*e*_*μ*, *S*=*2N*_*e*_*s*, *μ* is the mutation rate, and *s* is the selection coefficient [87]. The time for the emergence of *r* new alleles at the same locus is given by the formula $T_{r}=\frac{1}{2M}\overset{j=r}{\underset{j=1}{\sum }}\frac{1-e^{-\frac{2S}{j\left(j+1\right)}}}{1-e^{-\frac{S}{\mathrm{jN}}}}$ [EQ.3] in the unit of *2N*_*e*_[87]. To predict how the genomic diversity of the Lyme pathogen would be affected by the on-going population expansion of *B*. *burgdorferi* s.s. in North America [88,89], we simulated genome evolution under a model of immune escape and frequent recombination (described in Methods) [66]. Consistent with the above theoretical expectations, the steady-state sequence diversity at immune-escape loci increases proportionally with the population size (Figure 8A) and the number of distinct genomic lineages increases correspondingly (Figure 8B). These theoretical and simulation analyses may be used to estimate key population-genetic parameters of the Lyme disease endemics such as the effective population size, time since colonization, and time for emergence of new genomic groups. For example, the mutation parameter of *B*. *burgdorferi* s.s. in Northeastern U.S. could be estimated from nucleotide diversity at the ribosomal RNA spacer (IGS) loci, because simulations on the effects of intra-genic recombination showed a gradual decay of *f*_*s*_ towards one (i.e., neutral expectation) at loci with increasing distances from the selection target [90]. Since *M*=*2N*_*e*_*μ*_*0*_=*0*.*025* at an IGS locus [34] and assuming a neutral mutation rate *μ*_*0*_=*1x10*^-*9*^, we obtained an estimate of *N*_*e*_=*1*.*3x10*^*7*^ for *B*. *burgdorferi* s.s. in the Northeastern U.S. If the selective advantage of a new allele is *s*=*1x10*^-*3*^ at *ospC*, then *S*=*N*_*e*_*s*=*1*.*3x10*^*4*^. Considering that there are currently about 20 *ospC* major alleles segregating in the Northeastern U.S., it could be estimated from EQ.3 that the time since its introduction from Europe is in about 0.5*N*_*e*_ generations, which is fifty thousand years if we assume *B*. *burgdorferi* replicates 100 generations per year. The rise of the first new *ospC* allele would take about 300 years based on EQ.2. Both of these time estimates would be considerably shortened by taking into consideration the fact that the intragenic recombination rate is three times the mutation rate [49].

## Conclusions

We have determined the genome sequences of 22 *B*. *burgdorferi* s.l. isolates. This information was used to generate SNP trees of the chromosome and the most conserved plasmids cp26 and lp54. These trees robustly show phylogenetic relationships among these isolates both within and among different species. Our results show that *B*. *burgdorferi* s. s. and *B*. “*finlandensis*” form a closely related group, as do *B*. *garinii* and *B*. “*bavariensis*”. *B*. *bissettii* is rather closely related to *B*. *burgdorferi* s.s., but *B*. “*finlandensis*” is the closest known outgroup of *B*. *burgdorferi* s.s. *B*. *afzelii* and *B*. *spielmanii* form a robust (but less closely related) group, and *B*. *valaisiana* is not especially closely related to any of the other species analyzed here. These findings are largely in agreement with previous studies using only a few sequences from each isolate, but they provide a statistically much more robust and quantitative description of these relationships. We also find that, despite fairly frequent within-population recombination, the *B*. *burgdorferi* s.s. isolates fall into four well-supported groups.

We conclude that the intra- and inter-specific pan-genome sizes of *B*. *burgdorferi* s.l. depend strongly on its phylogenetic history. By taking phylogenetic relatedness among pathogen genomes into consideration, phylogeny-guided pan-genome analysis removes sampling artifacts in traditional approaches based on genome numbers and yields robust predictions on the pan-genome sizes of pathogens as well as their rates of gene acquisition. *B*. *burgdorferi* s.l. has a highly stable genome, with one of the lowest gene-acquisition rates and one of the largest core genome among bacterial pathogens. Adaptive genome differentiation between and within *B*. *burgdorferi* s.l. species is driven mostly by copy-number and sequence variations rather than by gains and losses of lineage-specific virulence genes. Finally, we predict a rapid emergence of novel *ospC* groups in areas newly colonized by *B*. *burgdorferi* s.l. Due to a lack of molecular clock to calibrate the time scale of sequence evolution, however, it remains a challenge to estimate a time scale and the rate of such emergence of new genomic groups in local *B*. *burgdorferi* s.l. populations.

## Methods

### Provenance of the *Borrelia* isolates, propagation and DNA isolation

In order to select strains of *B*. *burgdorferi* s.s. with the highest level of genetic diversity, isolates were obtained from clinical and tick specimens and cultures from animals in the U.S. and Europe as previously described (Table 1) [34]. Spirochetes were cultivated at 34°C in complete BSK II medium (Sigma, St. Louis, Mo) and total genomic DNA was then isolated from 10 ml of low passage log-phase bacteria after centrifugation at 10,000 rpm for 30 min at 4°C. Pellets were washed twice with Tris-Cl buffer (10mM Tris pH7.5, 100 mM NaCl), and resuspended in 430 μl TES (10 mM Tris pH7.5, 100 mM NaCl, 10 mM EDTA). Subsequently, 10 μl of freshly prepared lysozyme (50 mg/ml), 50 μl Sarkosyl (10%), and 10 μl proteinase K (10 mg/ml) were then added and the mixture was incubated at 50°C overnight prior to RNase treatment. Following incubation, DNA was extracted with phenol/chloroform and chloroform, precipitated with ethanol, and finally resuspended in TE buffer (1 mM Tris pH7.5, 1 mM EDTA).

### Multilocus sequencing analysis (MLSA)

PCR amplification followed by DNA sequencing across 64 *B*. *burgdorferi* s.l. isolates was performed on 6 chromosomal housekeeping genes (*gap*, *alr*, *glpA*, *xylB*, *ackA*, *tgt*), *ospC* and the IGS locus as previously described [34]. Sequences for these 6 housekeeping genes and the IGS locus were deposited to Genbank under the following accession numbers: *gap*, KC416567 - KC416611; *alr*, KC416477 - KC416521; *glpA*, KC416432 - KC416476; *xylB*, KC416316 - KC416360; *ackA*, KC416522 - KC416566; *tgt*, KC416361 - KC416405; and *rrs*-*rrlA*, KC416406 - KC416431.

The 6 housekeeping genes and the IGS sequences were used to infer the overall within- and between-species phylogeny among *B*. *burgdorferi* isolates [37,41]. Orthologous sequences from the *Borrelia hermsii* DAH genome (NCBI BioProject PRJNA29637) were collected for the purpose of rooting the MLST phylogenetic tree. The concatenated DNA sequences at the 7 loci were aligned using ClustalW [91]. Two approaches, a Bayesian method with codon site-specific evolutionary rates using MrBayes [92] and the other maximum likelihood method with 100 bootstrapped alignments using DNAML in PHYLIP [93], were employed for phylogenetic reconstruction based on concatenated sequences. Branch supports were measured by the posterior probabilities in the Bayesian method and the bootstrap values in the maximum likelihood method. We selected 22 isolates representing major phylogenetic lineages for whole-genome sequencing to estimate the size and composition of the pan-genome of the *B*. *burgdorferi* s.l. species complex. Thirteen of these sequenced isolates represent major evolutionary lineages of *B*. *burgdorferi* sensu strico, a widely distributed pathogenic species causing Lyme disease across three continents (see below).

### Whole-genome shotgun sequencing: library construction, genome assembly and closure

All the *Borrelia* strains included in this study were sequenced as previously described [94] by the random shotgun method using Sanger DNA sequencing to an estimated 8-fold coverage. Unless otherwise noted in Table 1, all the plasmids were sequenced to closure, while some sequencing and physical gaps were left in the chromosomes. Briefly, one small insert plasmid library (2–3 kb) and one medium insert plasmid library (7–8 kb) were constructed for each strain and sequenced to ~5X and 3X coverage, respectively. Approximately 10,625 and 6,375 successful reads for the small and medium insert plasmid libraries were sequenced, representing a total of ~14 Mb of sequencing data for each strain. The sequences from the respective strains were assembled separately using a combination of the TIGR Assembler [95] and Celera Assembler [96]. All sequence and physical gaps in the plasmids were closed by editing the ends of sequence traces, primer walking or transposon-primed sequencing [97] on plasmid clones, and combinatorial PCR followed by sequencing of the PCR product. Pseudo-molecules for the draft sequences of the chromosomes and some of the plasmids (Table 1) were constructed using NUCmer [98] and BAMBUS [94,99] as previously described [94].

### Genome annotation and open reading frame (ORF) nomenclature

Genome annotation was performed using the JCVI Prokaryotic Annotation Pipeline (http://www.jcvi.org/cms/research/projects/prokaryotic-annotation-pipeline/overview/). Briefly, an initial set of open reading frames (ORFs) likely to encode proteins was identified by GLIMMER (http://ccb.jhu.edu/software/glimmer/index.shtml). ORFs that overlapped were inspected visually and, in some cases, removed. ORFs were searched against an internal non-redundant protein database, PANDA (Protein and Nucleotide Data Archive) as described previously for all JCVI genomes. PANDA is JCVI’s internal repository of non-redundant and non-identical protein and nucleotide data built periodically from public databases that include the latest protein sequences (e.g. GenBank (http://www.ncbi.nlm.nih.gov), PDB (http://www.rcsb.org/pdb/Welcome.do), UniProt (http://www.uniprot.org/) and the Comprehensive Microbial Resource database (http://www.tigr.org/CMR)). Two sets of hidden Markov models (HMMs) were used to determine ORF membership in families and superfamilies. These included 10,340 HMMs from PFAM version 23.0 (http://pfam.sanger.ac.uk/) and 3,603 HMMs from TIGRFam version 8.0 (http://www.jcvi.org/cms/research/projects/tigrfams/overview/). TOPPRED was used to identify membrane-spanning domains in proteins.

In an effort to improve the accuracy and consistency of the annotation of the chromosomal genes, curation of the JD1, N40 and 297 genome annotation was performed in parallel. The B31 genome annotation was also updated in the process. ORFs ≤50 codons were not annotated, and those in the 51–100 range were not annotated unless they are intact in all three of the chromosome since the 297 chromosome sequence was not determined. Two chromosomal ORFs, *B31*_*0771a* and *B31*_*0838a*, were identified that were not recognized in the original annotation of the B31 chromosome. Including these comparisons in the analysis results in the annotation of significantly fewer short ORFs as potentially functional genes; many of those not included in the present analysis were previously suspected to be spurious gene identifications and not functional genes [57]. We thus identify 815 putative protein coding genes in the constant region of these chromosomes. These 815 putative genes as well as the tRNA, tmRNA and rRNA genes are all present and in identical locations in all *B*. *burgdorferi* chromosomes. Comparison of the predicted ORFs in the constant regions of the *B*. *burgdorferi* B31, N40 and JD1 chromosomes identified twenty ORFs in which one is disrupted by an in-frame stop or frameshift relative to the other two strains (Additonal file 1: Table S1). Some of these differences may be the result of sequencing errors; *e*.*g*., *B31*_*0078* and *0079* are joined as one gene in Genbank Accession AF492471, and are now annotated as one gene in these four genomes.

*Borrelia* researchers have usually used the “locus tags” of the strain B31 genome GenBank annotation [57,86] as names for genes and their encoded proteins. Thus, according to bacterial convention, the B31 chromosomal genes have been named ”*bb0xxx*” (lower case and italicized) in ascending order from *bb0001* upward across the chromosome. The B31 plasmid locus tag names are similar but have the form ”*bb*$*xx*” in which ”$” is a letter code denoting which plasmid type carries the gene (*e*.*g*., *a74* encodes protein A74 and lies on lp54, *s09* lies on cp32-3, *etc*.). Increased genome sequencing forces the use of more complex locus tags. For example, BbuJD1_Axx for strain JD1 plasmid lp54 with its letter code of A. We suggest the use the form “strain name_locus tag letter and/or number only” for gene names when it is important to delineate their strain source (*e*.*g*., *B31*_*0843* for a B31 chromosomal gene, and ”*JD1*_$##” for a JD1 plasmid gene with plasmid letter code). In the different genomes, the same locus tag numbers in the chromosome, cp26 and lp54 usually indicate orthology of the corresponding genes; however, organizational differences in the other plasmids made this system unworkable so the same locus tag numbers on these replicons do *not* indicate orthology.

### Whole-chromosome phylogenetic analysis and percent identity calculations

The chromosomal sequences from 26 *Borrelia* sp. were aligned with Mugsy [100], which incorporates MUMmer [98,101] and SeqAn [102]. Mugsy performs fast multiple alignments of closely-related whole genomes without requiring a reference sequence. It is robust in identifying a rich complement of genetic variation including duplications, rearrangements, and large-scale gain and loss of sequence. The Mugsy computes produced an output composed of blocks of conserved, aligned sequences between species in a MAF file format. Blocks were then joined together and converted to a multifasta file with the *bx*-*python* toolkit (http://bitbucket.org/james_taylor/bx-python/wiki/Home). The resulting alignment of twenty-two *Borrelia burgdorferi* s.l. chromosomal conserved blocks of sequence was 906,966 bp long including small indels. This alignment was further processed, and columns with gaps in any one genome were removed, resulting in an 843,710 bp chromosomal core sequence alignment. Considering that the common region of the *Borrelia* chromosome is about 903 kp long, our chromosome core sequence alignment incorporates 95.4% of the potential genetic information of the chromosomes, and it provides a fast and accurate estimate of the substitution differences that have accumulated between the unambiguously homologous sequences that are present in *all* of the genomes being compared.

An approximately-maximum-likelihood phylogenetic tree (Jukes-Cantor + CAT model) was subsequently inferred from the final Mugsy alignment using FastTree2 [103] with one thousand bootstrap replicates and a generalized time-reversible model. The Mugsy alignment was also used to calculate the percentages of nucleotide similarities and differences between the chromosomal blocks of conserved core sequences, using the *infoalign* program from the EMBOSS software analysis package [104].

### Core genes, gene discovery and pan-genome computations

Core-genome and pan-genome calculations were performed as previously described by Tettelin and colleagues [69]. Briefly, estimations of core genes, new genes, and pan-genome size were performed using all-versus-all WU-BLASTP and all-versus-all WU-TBLASTN searches according to W. Gish (http://blast.wustl.edu) [105]. The results from these two sets of searches were combined such that the TBLASTN searches prevented missing gene annotations from producing false negatives. Sequence gaps in the draft chromosomes (Table 1) were sufficiently small that they contained few, if any, chromosomal genes. Hits were filtered such that homologues were defined as having 50% sequence similarity over at least 50% of the length of the protein. The determination of core genes and strain-specific genes depends on the number of genomes included in the analysis. The number (N) of independent measurements of the core and strain-specific genes present in the n^th^ genome is N = S/((n-1)!·(S-n)!), where S is 13 (*B*. *burgdorferi* s.s. ) and 21 (*B*. *burgdorferi* + other *Borrelia* species). A random sampling of 1000 measurements for each value of n was calculated to reduce the number of required computations. The numbers of core and strain-specific genes for a large number of sequenced isolates were extrapolated by fitting the exponential decaying functions *F*_*c*_(*n*) = κ_*c*_*exp*(−*n*/τ_*c*_) + *tg*_*c*_(θ) and *F*_*n*_(*n*) = κ_*n*_*exp*(−*n*/τ_*n*_) + *tg*_*n*_(θ), respectively, to the mean number of conserved and strain-specific genes calculated for all strain combinations. *n* is the number of sequenced strains, and κ_*c*_, τ_*c*_, κ_*n*_, τ_*n*_, *tg*_*c*_(θ), and *tg*_*n*_(θ) are free parameters. *tg*_*c*_(θ) and *tg*_*n*_(θ) represent the extrapolated number of core and strain-specific genes, assuming a consistent sampling mechanism and a large number of completed sequences. The pan-genome itself represents an estimation of the complete gene pool based on the set of genomes analyzed and was computed in triplicates. In this case, a sample of at most 1,000 combinations for each value of *n* was taken and the total number of genes, both shared and strain specific, was calculated. A power law regression was then fitted to estimate the total number of genes accessible to the subsets of tested genomes or the pan-genome, using the median values at each *n*. The least-squared model fitting was performed using the “*nls*” (Nonlinear Least Squares) function in R (http://www.r-project.org/).

Regression of the pan-genome size against the total tree length allows for an estimation of the rate of gene acquisition: *Ω*_*n*_*=Ω*_*0*_*+ωT*_*n*_, where *ω* is the rate of acquisition of new genes and *Ω*_*n*_ and *T*_*n*_ are, respectively, the pan-genome size and the total tree length of *n* sampled genomes [77]. We calculate the tree length of sampled genomes based on the chromosome SNP tree by using customized Perl scripts based on the BioPerl [106] programming library. We used the R statistical package for linear regression analysis.

### Jaccard Orthologous Clustering (JOC) analysis

Jaccard orthologous clustering was used to cluster proteins from the different *Borrelia* genomes analyzed in this study, in order to identify orthologous genes. Jaccard clustering was performed using the Sybil software package [72,107], available at Sourceforge (http://sybil.sourceforge.net/) and implemented at the Institute for Genome Sciences using the Ergatis bioinformatics workflow [108]. The following parameters were used: Jaccard coefficient = 0.6 and minimum BLASTP percent identity threshold = 80%.

### Single-nucleotide polymorphism discovery and analysis

Single-nucleotide polymorphisms were identified in pair-wise genome comparisons between the predicted genes on the closed chromosome, as well as linear plasmid lp54 and circular plasmid cp26, of *B*. *burgdorferi* strain B31 and the corresponding chromosome and plasmids of 23 strains of *Borrelia* (see Table 1) using MUMmer [98]. We note that all of the other plasmids have suffered enough inter-plasmid recombination to make assignments of orthologs challenging and therefore were not included in this analysis. By mapping the position of the SNP to the annotation in the reference strain B31 genome, it was possible to determine the effect on the deduced polypeptide and classify each SNP as synonymous (sSNPs) or non-synonymous (nsSNPs). The SNP data set was curated to include only SNPs in MUMmer alignments with at least 70% identity. Positions within repeats and regions with greater than 5% gap characters were excluded from the analysis. All stop codons in the aligned sequences were identified, and the corresponding positions were removed from all sequences. sSNPs and nsSNPs for each *Borrelia* strain compared to the reference B31 were then concatenated to form “SNP pseudosequences”, which were used to generate a phylogenetic tree using the HKY93 algorithm [109] with 500 bootstrap replicates. The Geneious software package (http://www.geneious.com) and SplitsTree4 (http://www.splitstree.org/) were used for visualization.

The Phylogenetic Analysis by Maximum Likelihood (PAML) programs package, and more specifically the codeml program, was used for *K*_*A*_/*K*_*S*_ analyses. To estimate a single *K*_*A*_/*K*_*S*_ ratio averaged over all lineages and all sites, the basic model of Goldman and Yang was used [110].

### Simulation of genome evolution

We used SimBac (http://sourceforge.net/projects/bacsim/), a software package for simulating bacterial genome evolution, to predict the total number of genomic lineages in local *B*. *burgdorferi* s.s. populations [66]. These simulations allowed us to explore how deeply natural populations need to be sampled in order to fully account for their total genomic diversity, whether the genomic diversity is stable or increases over time, and how genomic diversity is influenced by recombination, natural selection, and population expansion. Briefly, the simulated bacterial population initially contained 1,000 identical genomes, each of which consisted of 6 protein-coding genes. In mimicking genome evolution driven by variations of major surface antigens (e.g., *ospC*), we designated one gene (the “FDS” locus) to be under the influence of positive natural selection, in which amino-acid replacement mutations were preferred in a negative-frequency-dependent fashion. Amino-acid replacement mutations at all other gene loci lowered the genome fitness, simulating housekeeping genes in a bacterial genome. We kept the population size constant and let the population evolve for 10,000 to 20,000 generations. During each none-overlapping generation, individual genomes were subject to random uniform mutations and gene conversion. The genetic structure of the population was characterized using average pairwise nucleotide differences (*π*), coalescent tree, and the lineage-through-time (LTT) plot. The LTT plot tracks the number of evolutionary lineages of a coalescence tree at regular time intervals and, thereby, helps to visualize the rate of lineage diversification over time [111]. The coalescence tree of a *B*. *burgdorferi* s.s. population under balancing selection is characterized by elongated internal branches and compressed terminal branches relative to a neutrally derived coalescence tree. Such long internal branches correspond to major evolutionary lineages in the population. Since long internal branches appear as a period of stasis on a LTT plot, we use the height of such stasis as an estimate of the total number of major lineages in a population. To explore the effect of sampling to estimation of genetic diversity, we sampled 100 individuals from the final stabilized population. To estimate the rate of increase of genetic diversity over time, we sampled 50 individuals every 500 generations. Lastly, we varied population size to predict the effect of population expansion to the genomic diversity in *B*. *burgdorferi* s.l. populations.

## Abbreviations

s.s.: Sensu stricto; s.l.: Sensu lato; Osp: Outer-surface protein; Pfam: Paralogous family; SNP: Single nucleotide polymorphism; MLST: Multi-locus sequence typing; JOC: Jaccard orthologous clustering.

## Competing interests

The authors declare that they have no competing interests.

## Author’s contributions

Conceived and designed the experiments: EFM, BLL, SRC, WGQ, JJD, SES and CMF. Performed the experiments and genome analyses: EFM, WGQ, SRC, JFB, YX, EFD, DRR, BLC, PEP, YAH and LCV. Wrote the paper: EFM, WGQ and SRC. All authors read and approved the final manuscript.

## Supplementary Material

Additional file 1: Table S1 *B*. *burgdorferi* s.s. chromosomal open reading frame differences. Click here for file

Additional file 2: Table S2 *B*. *burgdorferi* s.s chromosomal indels >25 bp. Click here for file

Additional file 3: Table S3 Chromosome structural differences between *B*. *burgdorferi* B31 and *B*. *afzelii* PKo and ACA-1. Click here for file

Additional file 4: Figure S1 *B*. *burgdorferi* sensu lato chromosomal differences in the gene *0522*–*0524* region. Translational six-reading frame diagrams are shown for the gene *B31*_*0522*-*0527* region and homologs in other isolates. Translation is left to right in top three frames where the open reading frames are red, and right to left in bottom three, where the open reading frames are yellow. Genes with apparently broken open reading frames are indicated in green (it is not known if the B31_*0522* frame is broken or if this represents a sequencing error). Long vertical lines in each frame represent stop codons and short vertical lines indicate methionine codons. Numbering of bps starts at the beginning of the *grpE* (*519*) gene. Click here for file

Additional file 5: Table S4 Orthologous gene clusters. Each number in columns 3 through 24 represents the number of proteins for each genome for a given cluster. Click here for file

Additional file 6: Table S5 Species-specific ORFs. Click here for file

Additional file 7: Table S6 *K*_*A*_, *K*_*S*_, and *K*_*A*_/*K*_*S*_ values of individual ORFs for both within- and between-species comparisons. Click here for file
