# Supplementary material for: Inter- and intra-specific pan-genomes of Borrelia burgdorferi sensu lato: genome stability and adaptive radiation
Source: BMC Genomics. 2013 Oct 10;14:693. doi: 10.1186/1471-2164-14-693 (PMC3833655; doi:10.1186/1471-2164-14-693)
Supplement: Additional file 1: Table S1 — B. burgdorferi s.s. chromosomal open reading frame differences. [file 1471-2164-14-693-S1.pdf]

Supplementary Table 1: *B. BURGDORFERI* CHROMOSOMAL OPEN READING FRAME DIFFERENCES[a]

---

1. *B31_0003* Different frameshifts in B31 and N40 relative to the *apparently* full-length JD1 ORF.
2. *B31_0021* Encodes a putative S-adenosylmethionine:tRNA ribosyltransferase-isomerase, contains an internal frameshift in B31, a frameshifting 157 bp deletion JD1 and the 157 bp deletion and an in-frame stop codon in N40. This gene is intact in some *B. burgdorferi* strains such as ZS7 as well as *B. garinii* PBi and *B. Afzelii* PKo.
3. *B31_0078* and *B31_0079* – B31 ORFs are fused into one gene in strains N40 and JD1. This fused gene is homologous to the *lolC* family which encodes part of an ABC transporter that is essential for lipoprotein export in other species W. Zückert and J. Liu (pers. comm.; Accession No. AF492471) have shown that there is an extra A at chromosomal bp 75013 in their B31 cultures, suggesting this difference is due a sequencing error in the original genome sequence.
4. *B31_0140* Frameshift difference in B31 relative to the others.
5. *B31_0205* Frameshift difference in B31 that extends its open reading frame relative to the others.
6. *B31_0357* B31 ORF has an internal in-frame stop codon relative to the others.
7. *B31_0381* B31 ORF has a frameshift very near the 3'-end of the gene relative to the others.
8. *B31_0411* B31 ORF has two frameshift sequence differences relative to the others.
9. *JD1_0427* JD1 ORF has an internal in-frame stop codon relative to the others.
10. *B31_0511* B31 ORF has two frameshift sequence differences relative to the others.
11. *B31_0516* Frameshift difference in B31 relative to the others.
12. *B31_0522* B31 ORF has 2 frameshift differences relative to the others ; predicted to be an NAD+ synthetase (Supplemental Figure 1).
13. *JD1_0524* In JD1 a frameshift shortens this ORF (sugar phosphatase) relative to B31 and N40 (Supplemental Figure 1).
14. *B31_0530* B31 ORF has a frameshift difference relative to the others.
15. *B31_0532* B31 ORF has a frameshift difference relative to the others.
16. *B31_0634* *recC ; i*n B31 a frameshift sequence difference shortens this ORF relative to orthologues in the other genomes.
17. *JD1_0701* JD1 ORF has an internal frameshift relative to the other genomes.
18. *B31_0710* and *B31_ 0711* *dnaG ;* in B31 a frameshift sequence difference shortens this ORF relative to orthologues present in the other genomes.
19. *B31_0751* This B31 ORF bas an internal in-frame stop codon relative to the others.
20. *N401_0799* The JD1 and N40 ORFs have an internal in-frame stop codon relative to the others.
21. *B31_0815* This B31 ORF has a frameshift difference relative to the others.

PREVIOUSLY UNRECOGNIZED PUTATIVE GENES

(1) Gene *B31_ 0771a* has been added to B31 genome annotation.

(2) Gene *B31_ 0838a* has been added to B31 genome annotation.

Supplementary Table 1 Footnote:

a. Twenty-one reading frame differences were found among the B31, N40 and JD1 chromosomal genes and these are listed in the table. In most cases, it not known if these differences represent authentic frame-disrupting mutations, outside of genes due to inaccurate gene assignment, or sequencing errors. Identification of a frame-disrupting sequencing error in *B31_ 0078* in B31 (above), frameshift differences in B31 that shorten important genes *recC* (*B31_ 0634*) and *dnaG* (*B31_ 0710*), and the fact that the B31 genome, which was determined before recent advances in sequencing accuracy, harbors most of these disruptions, combine to suggest that the latter is likely true in a number of these cases.
