# Supplementary material for: Inter- and intra-specific pan-genomes of Borrelia burgdorferi sensu lato: genome stability and adaptive radiation
Source: BMC Genomics. 2013 Oct 10;14:693. doi: 10.1186/1471-2164-14-693 (PMC3833655; doi:10.1186/1471-2164-14-693)
Supplement: Additional file 2: Table S2 — B. burgdorferi s.s chromosomal indels >25 bp. [file 1471-2164-14-693-S2.pdf]

Supplementary Table 2 : *B. burgdorferi* s.s chromosomal indels >25 bp

Strain	Indel 1	2	3	4
64b	+fs	7	3	12
72a	+	5	4	11
94a	+fs	6	4	11
118a	+fs	7	4	11
156a	–fs	8	5	11
29805	+	6	3	11
B31	+fs	7	5	12
Bol26	+	7	4	11
CA-11.2A	+fs	6	5	11
JD1	–fs	6	5	11
N40	– fs,st	7fs	3	10
WI91-23	–fs	7fs	4	11
ZS7	+	5	4	12

Footnotes for Supplementary Table 2

1 "–" indicates a 157 bp deletion in gene *bb0021*
2 Number of 54 codon repeats in gene *bb0210* (*lmp1*)
3 Number of 20 codon repeats in gene *bb0546*
4 Number of 11 codon repeats in gene *bb0801*
fs Contains frameshift in DNA sequence
st Contains in-frame stop codon
