# Supplementary material for: Inter- and intra-specific pan-genomes of Borrelia burgdorferi sensu lato: genome stability and adaptive radiation
Source: BMC Genomics. 2013 Oct 10;14:693. doi: 10.1186/1471-2164-14-693 (PMC3833655; doi:10.1186/1471-2164-14-693)
Supplement: Additional file 3: Table S3 — Chromosome structural differences between B. burgdorferi B31 and B. afzelii PKo and ACA-1. [file 1471-2164-14-693-S3.pdf]

Supplementary Table 3 : Chromosome structural differences between *B. burgdorferi* B31 and *B. afzelii* PKo and ACA-1

**Difference relative to B31**	**B31**	**PKo**	**ACA-1**
bb0138 present	+	–	–
No. of repeats in *bb0210*	7	5	5
bb0223 present	+	–	–
Insert in *bb0309*	–	44 bp	44 bp
bmpA gene duplication	–	+	+
16S rRNA gene duplication	–	+[a]	+[a]
Insert between *bb0456* & *0457*	–	56 bp	56bp
Deletion between *bb0472* & *0473*	–	171 bp	171 bp
Deletion between *bb473* & *0475*	–	~145 bp[a,b]	~145 bp[a,b]
Indel between *bb522* & *0524*	–	~250 bp[b]	~250 bp[b]
No. of repeats in *bb0546*	5	3	4
Insert in *bb0744* (p83/p100)	–	~118 bp[b]	~118 bp[b]
Deletion in *bb0749*	–	143 bp	143 bp
Insert between *bb0796 & 797*	–	~330 bp[b]	~330 bp[b]
No. of repeats in *bb0801*	12	6	12

Footnotes for Table S3

\+ Present

– Not present

a. *bb0423, bb0424* and *bb0474* in the original B31 annotation were small ORFs that are not open in other *burgdorferi* genomes, so they have been removed from that annotation. All three of these regions are affected in PKo and ACA-1.

b. Rearrangement is more complex than simple deletion; value depends on precise choice of endpoints.
