# Supplementary figures and images for: Inter- and intra-specific pan-genomes of Borrelia burgdorferi sensu lato: genome stability and adaptive radiation
Source: BMC Genomics. 2013 Oct 10;14:693. doi: 10.1186/1471-2164-14-693 (PMC3833655; doi:10.1186/1471-2164-14-693)

**Supplementary Figure 1:** *B. burgdorferi* chromosomal differences in the gene0522-0524 region.

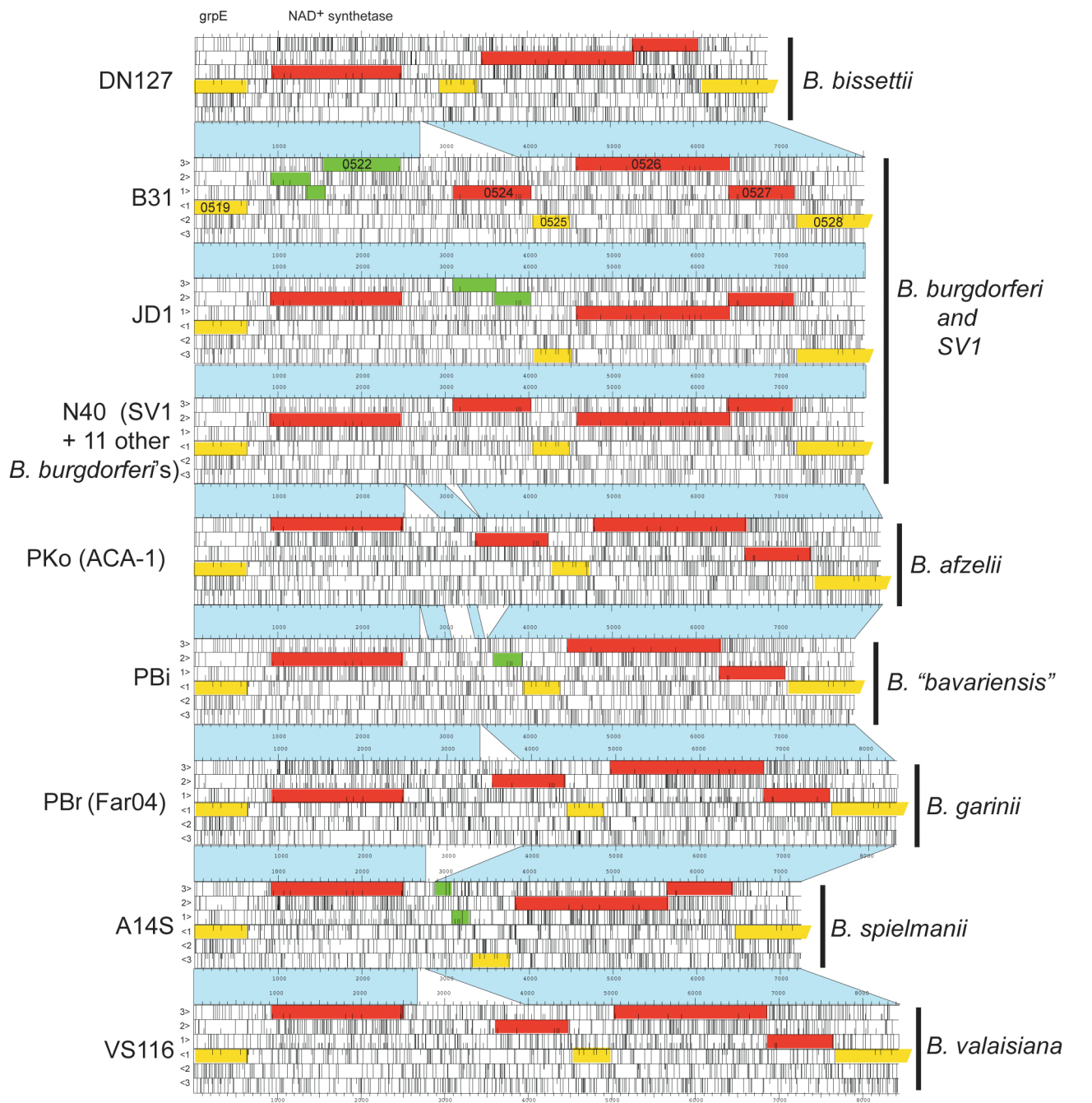

Supplement: Additional file 4: Figure S1 — B. burgdorferi sensu lato chromosomal differences in the gene 0522–0524 region. Translational six-reading frame diagrams are shown for the gene B31_0522-0527 region and homologs in other isolates. Translation is left to right in top three frames where the open reading frames are red, and right to left in bottom three, where the open reading frames are yellow. Genes with apparently broken open reading frames are indicated in green (it is not known if the B31_0522 frame is broken or if this represents a sequencing error). Long vertical lines in each frame represent stop codons and short vertical lines indicate methionine codons. Numbering of bps starts at the beginning of the grpE (519) gene. [file 1471-2164-14-693-S4.pdf]
